# Catabolic System of Syringic Acid, a Key Intermediate of Lignin-Derived Aromatic Compounds, via a Novel Linear Pathway in *Pseudomonas* sp. NGC7

**DOI:** 10.1021/acs.jafc.5c04544

**Published:** 2025-07-16

**Authors:** Zen Ookawa, Yudai Higuchi, Masaya Fujita, Tomonori Sonoki, Naofumi Kamimura, Eiji Masai

**Affiliations:** † Department of Materials Science and Bioengineering, 52756 Nagaoka University of Technology, Nagaoka, Niigata 940-2188, Japan; ‡ Faculty of Agriculture and Life Science, 12800 Hirosaki University, Hirosaki, Aomori 036-8561, Japan

**Keywords:** *Pseudomonas*, lignin, syringic acid, catabolic pathway, transcriptional regulation, formaldehyde detoxification, 2-pyrone-4, 6-dicarboxylic acid

## Abstract

Syringic acid (SA) is a key intermediate in the bacterial catabolism of syringyl lignin-derived aromatic compounds. However, bacterial SA catabolism remains largely unknown. Here, we investigated the SA catabolic system in *Pseudomonas* sp. NGC7, which catabolizes various lignin-derived aromatic monomers. Pathway analysis and gene disruption studies revealed that SA undergoes O demethylation by VanA1B1 to 3-*O*-methylgallic acid, which is subsequently catabolized through a linear pathway, involving MgaAB, MgaC, MgaD, GalD, GalB, and GalC into the TCA cycle, unlike the branching pathway found in sphingomonad strains. *vanA1B1*, *mgaAB*, *mgaC*, and *mgaD* constitute an operon whose transcription is regulated by the GntR-type transcriptional repressor VanR2. In contrast, *galBCD* constitutes an operon with the formaldehyde dehydrogenase gene *fdhA2*, and this operon is regulated by the LysR-type transcriptional activator GalR. FdhA2 is involved in the detoxification of formaldehyde, a byproduct of SA catabolism. These findings contribute to the valorization of syringyl lignin-containing biomass.

## Graphical abstract

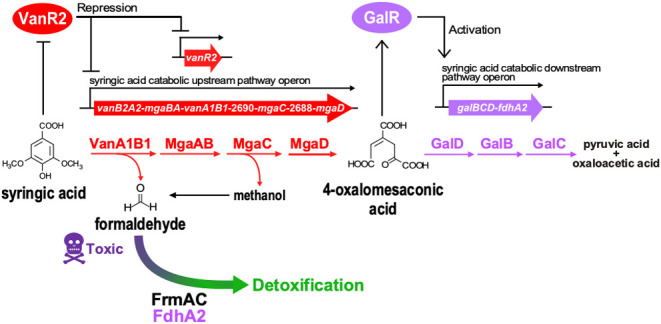

## Introduction

Lignin, a major component of plant cell walls, is the most abundant aromatic polymer in nature. To achieve a carbon neutral society, lignin has garnered significant attention as a potential alternative feedstock for petrochemicals, which are in high demand.^1^ Recently, biological funneling,^2^ a process in which various aromatic compounds obtained by the chemical depolymerization of lignin are converted into single polymer-building blocks, such as *cis*,*cis*-muconic acid,^3^ 2-pyrone-4,6-dicarboxylic acid (PDC),^4^ and β-ketoadipic acid,^5^ through microbial conversion, has attracted attention. Therefore, the need to elucidate bacterial catabolic systems for lignin-derived aromatic compounds and the transcriptional regulation of the genes involved has increased.

Lignin is formed by the oxidative coupling of three monomers: coniferyl alcohol (guaiacyl [G] type), sinapyl alcohol (syringyl [S] type), and *p*-coumaryl alcohol (*p*-hydroxyphenyl [H] type).^6^ Lignin monomer composition differs depending on the plant species; softwood lignins mainly consist of G-type units, whereas hardwood lignins are mainly composed of S-type and G-type units. Lignin biodegradation proceeds via the depolymerization of lignin polymer by fungi and the subsequent mineralization of the resulting low-molecular-weight aromatic compounds by bacteria.^7–9^ In bacteria, G- and S-type lignin-derived aromatic compounds are catabolized to vanillic acid (VA) and syringic acid (SA), respectively. VA and SA are subsequently converted by *O*-demethylases to protocatechuic acid and 3-*O*-methylgallic acid (3MGA), which are then further catabolized.^10,11^ Therefore, the O demethylation of VA and SA represents a key step in lignin biodegradation. The catabolic systems of lignin-derived aromatic compounds have been extensively studied in strains such as KT2440,^12–14^ SYK-6,^10,11^ DSM 12444,^15,16^ and RHA1.^17^ In SYK-6 and DSM 12444, LigM and DesA are responsible for the O demethylation of VA and SA, respectively, which require tetrahydrofolate as a cofactor.^16,18,19^ On the other hand, in various Gram-negative and Gram-positive bacteria, such as *Pseudomonas* and *Rhodococcus* strains, the O demethylation of VA is carried out by a two-component monooxygenase, VA *O*-demethylase (VanAB), which requires NAD­(P)H as a coenzyme.^20–23^ VanAB consists of VanA, an oxygenase component containing a Rieske [2Fe-2S] domain, and VanB, a ferredoxin oxidoreductase component. VanB transfers electrons from NAD­(P)H to VanA, which in turn delivers these electrons to the oxygen molecule in its active site, forming high-valent iron-oxo species. This species reacts with the methoxy group at the 3-position of the aromatic ring of VA to insert an oxygen atom into the methyl C–H bond, which results in the release of the methyl moiety as formaldehyde (HCHO). Consequently, the O demethylation of VA produces protocatechuic acid along with HCHO as a byproduct.^24,25^ VanAB of *Pseudomonas* sp. HR199,^24^ KT2440,^26^ R1,^27^ pv citri 306,^28^ and *Streptomyces* sp. NL15-2K^29^ are known to convert both SA and VA. With respect to the SA catabolic pathway and the genes involved, pathways in which DesA is responsible for the O demethylation of SA to 3MGA have been identified in SYK-6 and DSM 12444 ^16,19^ (Figure 1). 3MGA is subsequently catabolized via three distinct branched pathways: (i) aromatic ring cleavage to form PDC, (ii) aromatic ring cleavage to form 4-carboxy-2-hydroxy-6-methoxy-6-oxohexa-2,4-dienoic acid (CHMOD), and (iii) O demethylation to form gallic acid.^16,30,31^ PDC and gallic acid are further converted to 4-oxalomesaconic acid (OMA) by hydrolysis and aromatic ring cleavage, respectively. In contrast, CHMOD undergoes hydrolysis and isomerization to form OMA, or nonenzymatic cyclization to generate PDC. OMA is subsequently converted to pyruvic acid and oxaloacetic acid, which eventually enter the tricarboxylic acid (TCA) cycle. In contrast, the SA catabolic pathways involving O demethylation by oxygenases were studied in and others, mainly by Dagley and co-workers between 1970 and 1990.^32–35^ However, the genes involved in this catabolic pathway remain unidentified.

**Figure 1:**
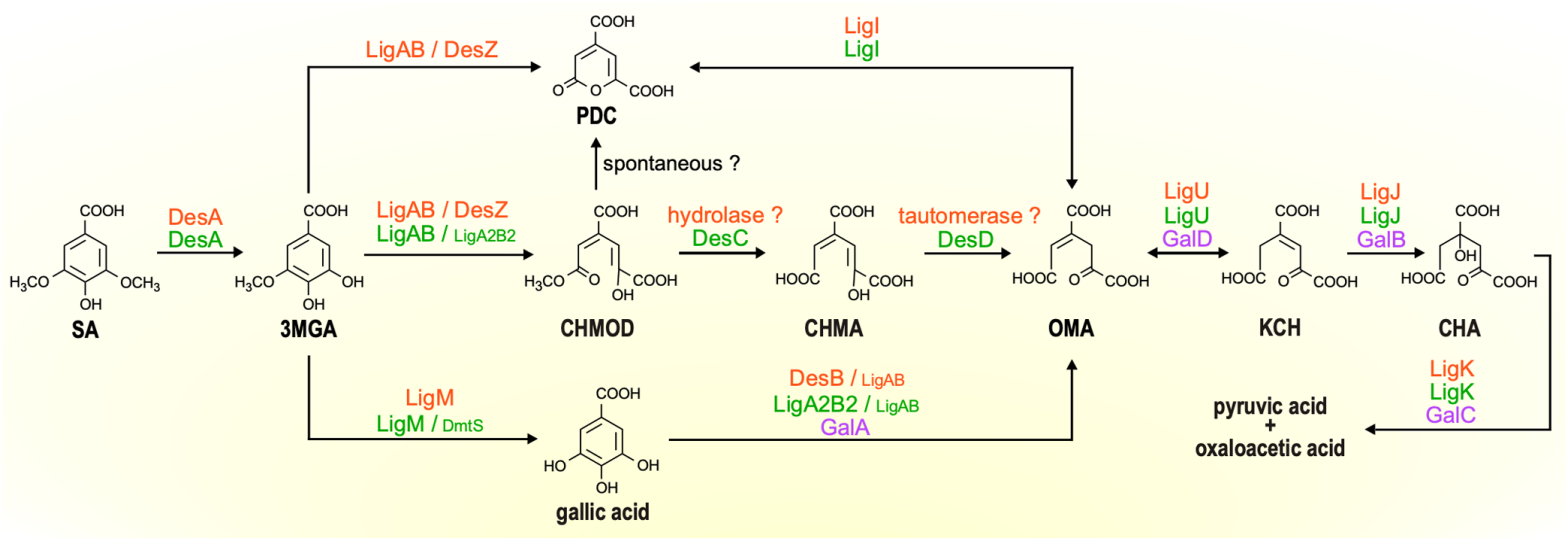
Bacterial SA catabolic pathways. SA catabolic enzymes from SYK-6 and DSM 12444 and gallic acid catabolic enzymes from KT2440 are indicated by orange, green, and purple, respectively. Abbreviations: SA, syringic acid; 3MGA, 3-*O*-methylgallic acid; PDC, 2-pyrone-4,6-dicarboxylic acid; CHMOD, 4-carboxy-2-hydroxy-6-methoxy-6-oxohexa-2,4-dienoic acid; CHMA, 4-carboxy-2-hydroxymuconic acid; OMA, 4-oxalomesaconic acid; KCH, 2-keto-4-carboxy-3-hexenedioic acid; CHA, 4-carboxy-4-hydroxy-2-oxoadipic acid. Enzymes: DesA, H_4_folate-dependent SA *O*-demethylase; LigAB, protocatechuic acid 4,5-dioxygenase α and β subunit; LigA2B2, aromatic ring-opening dioxygenase α and β subunit; DesZ, 3MGA 3,4-dioxygenase; LigM, H_4_folate-dependent VA/3MGA *O*-demethylase; DmtS, *O*-demethylase; LigI, PDC hydrolase; DesC and DesD, CHMOD methylesterase; DesB and GalA, gallic acid dioxygenase; LigU and GalD, OMA tautomerase; LigJ and GalB, KCH hydratase; LigK and GalC, CHA aldolase.

*Pseudomonas* strains have attracted attention as a platform for biological funneling because of their ability to catabolize various aromatic compounds,^36,37^ high resistance to oxidative stress,^38^ and well-established genetic manipulation systems.^38^ Among them, KT2440 is the most well-characterized bacterium with respect to aromatic compounds catabolic systems. The catabolic pathways of G- and H-type lignin monomers in KT2440 have been elucidated.^13,14^ While VanAB of KT2440 exhibits the conversion activity for SA in addition to VA,^26,39^ this strain is incapable of growing on S- type lignin-derived aromatic compounds such as SA. However, KT2440 can catabolize gallic acid from which methyl moieties of the methoxy groups of SA are eliminated, and its catabolic system has been revealed^12^ (Figure 1). In KT2440, the aromatic ring of gallic acid is cleaved by gallic acid dioxygenase (GalA) to form 4-oxalomesaconic acid (OMA), which is subsequently converted to pyruvic acid and oxaloacetic acid by GalD, GalB, and GalC, eventually entering the tricarboxylic acid (TCA) cycle.^12,40^ The gallic acid catabolism genes constitute the *galTAP* and *galBCD* operons, which include genes of the gallic acid inner membrane transporter (*galT*) and outer membrane porin (*galP*) genes. The transcription of these operons is regulated by the LysR-type transcriptional activator GalR.^12,41^

To date, transcriptional regulators for the expression of *vanAB* involved in VA catabolism have been reported, including the GntR-type repressor VanR from ATCC 17978,^42^ ADP1,^43^ and NA1000,^23^ the IclR-type activator VanR1 from PD630,^44^ and the PadR-type repressor VanR from ATCC 13032.^45^ In contrast, there have been no reports of a transcriptional regulatory system of *vanAB* responsible for SA catabolism. The transcription of *ligM* and *desA* in SYK-6 has been shown to be regulated by the MarR-type repressor DesR and the IclR-type repressor DesX, with VA and SA as major effectors, respectively.^46,47^

Previously, our group isolated *Pseudomonas* sp. NGC7, which grows well on all types of lignin-derived aromatic monomers with H-, G-, and S-type structures as the sole carbon sources.^48^ NGC7 is a promising microbial chassis for the production of value-added chemicals from mixtures of aromatic compounds obtained by the chemical degradation of lignin.^49–51^ Uniquely, NGC7 harbors multiple homologues of *vanAB* (*vanA1B1*, *vanA2B2*, *vanA3B3*, *vanA4B4*, *vanB5*, and *vanB6*), with each VanA and VanB sharing 26–39 and 32–71% amino acid sequence identity, respectively. Among them, VanA4B4, which shares 94 and 89% amino acid sequence identity with VanA and VanB of KT2440, respectively, is responsible for the O demethylation of VA.^50^ In contrast, SA is suggested to be O demethylated by VanA1B1, which shares 39 and 31% amino acid sequence identity with VanAB of KT2440, respectively.^50^ To our knowledge, there are no reports of bacterial strains harboring multiple *vanAB* homologues, including a dedicated *vanAB* for SA catabolism, as observed in NGC7. Therefore, elucidating the SA catabolic system in NGC7 is anticipated to increase the understanding of the microbial catabolism of S-type lignin-derived aromatic compounds.

In this study, we investigated the SA catabolic system in NGC7, focusing on the catabolic pathway, transcriptional regulation, and HCHO detoxification. The SA catabolic pathway in NGC7 is linear, contrasting with the branched pathway reported for sphingomonads. FdhA2 is involved in the detoxification of formaldehyde, a byproduct of SA catabolism. Additionally, we found that a HCHO dehydrogenase FdhA2 is involved in the detoxification of HCHO, and that its coding gene constitutes an operon with SA catabolic genes and is coinduced during SA catabolism. The findings of this study expand our understanding of the diversity of bacterial SA catabolic pathways and contribute to the development of fundamental technologies for lignin valorization.

## Materials and Methods

### Growth Analysis

The strains and plasmids, PCR primers, and synthetic DNA fragments used are summarized in Supporting Information Tables S1–S3, respectively. Culture conditions, genetic manipulations, and mutant construction are described in Supporting Information. RNA-seq analysis of NGC7 was performed as described in Supporting Information. NGC7, the gene disruption mutants, and the complemented strains were precultured in LB. Cells were harvested, washed twice with MMx-3 medium, and resuspended in the same medium. The cell suspensions were then inoculated into MMx-3 medium containing 5 mM SA, 1 mM Glc, or a mixture of 1 mM Glc and 0.2, 0.5, or 1 mM HCHO to an OD_600_ of 0.1. Cultures were incubated at 30 °C in an Epoch 2 microplate reader (Agilent BioTek), and OD_600_ was measured continuously. SA degradation product was analyzed using high-performance liquid chromatography (HPLC) as described in Supporting Information.

### Reverse Transcription (RT)-PCR and Quantitative RT (qRT)-PCR

Total RNA was extracted from cells of NGC7 and the gene deletion mutants cultured in MMx-3 medium containing 5 mM Glc (noninducing condition) or 5 mM Glc +5 mM SA (inducing condition), following the same protocol as in RNA-seq analysis. RNA concentration was quantified using the QuantiFluor RNA System (Promega). cDNA was synthesized from 2 μg of total RNA using the SuperScript IV Reverse Transcriptase (Invitrogen) and random hexamer primers. Reverse transcriptase negative control was included for each sample to confirm the absence of genomic DNA contamination. Synthesized cDNA was purified using the NucleoSpin Gel and PCR Clean-up kit (Takara Bio). RT-PCR was performed using primers (Table S2) designed to amplify the internal regions of each gene (Table S2) with 0.5 μL of cDNA or total RNA as the template and the resulting PCR products were separated by 0.8% agarose gel electrophoresis. qRT-PCR analyses were performed as previously described using SYBR Green PCR Master mix and StepOne Real-Time PCR system (Applied Biosystems).^52^ 16S rRNA of NGC7 was used as the housekeeping gene for normalization to compare the transcript levels across samples.

### Promoter Assay

A fragment of the mNeonGreen gene, codon-optimized for translation in , with a ribosome-binding site fused 5′ upstream of mNeonGreen gene was synthesized (Table S3). The synthetic fragments were amplified by PCR and inserted into the XbaI site in pSEVA221 to construct pSEVAmNeon (promoter analysis vector). The upstream regions of *vanB2*, *vanR2*, and *galB* used in the promoter assay were amplified by PCR using the NGC7 genomic DNA as template or synthesized and inserted into the XmaI site upstream of the ribosome-binding site of pSEVAmNeon. The DNA fragments containing mutations in the target motif were amplified by PCR using primers with the designed mutations (Table S2). Because pSEVAmNeon uses kanamycin resistance as a marker, the kanamycin resistance gene mutant Δ*aph*, was used as the wild-type control for promoter assays. Gene deletion mutant cells harboring the promoter analysis plasmid were precultured in LB, washed twice with MMx-3 medium, and resuspended in the same medium. The cells were then inoculated into MMx-3 medium containing 5 mM Glc (noninducing condition), 5 mM SA, 5 mM Glc + 5 mM SA, 5 mM Glc + 5 mM VA, or 5 mM Glc + 5 mM 3MGA to an OD_600_ of 0.2. The cultures were incubated at 30 °C in a synergy H1 microplate reader (Agilent BioTek). The OD_600_ and mNeonGreen fluorescence (excitation wavelength, 506 nm; emission wavelength, 547 nm) were measured continuously during incubation. Promoter activity was calculated by dividing the relative fluorescence units (RFU) at the plateau phase by the OD_600_ (RFU/OD_600_).

### Electrophoretic Mobility Shift Assay (EMSA)

The DNA fragments used as probes were amplified by PCR from NGC7 genomic DNA or synthetic DNA using primers listed in Table S2. The PCR products were separated and extracted using 2% agarose gels. Preparation of purified VanR2 protein is described in Supporting Information. DNA–protein binding reactions were conducted at 20 °C for 30 min in a 10 μL reaction mixture containing 1.5 pmol/μL VanR2, 8 fmol/μL DNA probe, 0.1 μg/μL poly-[d­(I–C)], 0.01 μg/μL poly-l-lysine, 1 mM MgCl_2_, and binding buffer [20 mM HEPES, 1 mM EDTA, 10 mM (NH_4_)_2_SO_4_, 1 mM dithiothreitol, 0.2% (w/v) Tween 20, 30 mM KCl, pH 7.6]. To examine the association of VanR2 with effector molecules, SA or 3MGA was added to the reaction mixture at concentrations of 10, 20, 30, 40, and 50 mM. Samples were separated by electrophoresis on a 2% agarose gel (Agarose 21; Nippon Gene) using 0.5× Tris/Borate/EDTA (TBE) buffer. DNA was stained with SYBR Gold Nucleic Gel Stain (Invitrogen) and visualized under a 470 nm blue LED by Safe Imager (Invitrogen).

### Search for Homologues of SA Catabolism Genes

Homologues were searched using NCBI Protein BLAST with nonredundant protein sequence database under the following settings: (i) excluding uncultured/environmental sample sequences, (ii) more than 40% (*vanB1*) or 50% (*vanA1*, *mgaA, mgaB, mgaC, mgaD, galD, galB, galC, ligA, ligB, ligM, desZ, desB, ligI, ligU, ligJ, ligK, pmdD, pmdU, pmdE, pmdF, desC, desD, vanA, vanB, and galA*) amino acid sequence identity, and (iii) more than 70% query coverage. Homologues of *vanA1* and *vanB1* are listed in Supporting Information Data Sheets 1 and 2, respectively. Among the organisms possessing homologues of *vanA1* and *vanB1*, one representative strain was selected from each genus to search for other SA catabolism genes. A similar search was also conducted for all eight *Pseudomonas* strains harboring both *vanA1* and *vanB1* homologues. The organisms used in this analysis are listed in Supporting Information Data Sheets 3 and 4.

## Results

### Identification of the SA Catabolic Pathway

To predict SA catabolism genes in *Pseudomonas* sp. NGC7, we investigated genes whose expression was induced in the presence of SA by RNA-seq Genes adjacent to *vanA1B1* and *vanA2B2* were significantly upregulated, with induction levels reaching up to 1215-fold in the presence of SA (Table S4 and Figure 2A). On the basis of these findings, we constructed deletion mutants of these highly induced genes (Figure S1) and assessed their growth on SA. Additionally, a previous study reported that deletion mutants of *vanA1B1* and *vanA2B2* lost and reduced their ability to grow on SA, respectively.^50^ To confirm these observations, we re-evaluated the growth ability of these mutants on SA.^50^ The *vanA1B1*, PSN_2689, PSN_2693–2694, PSN_2700, PSN_2701, and PSN_2702 deletion mutants (Δ*vanA1B1*, Δ2689, Δ2693–2694, Δ2700, Δ2701, and Δ2702, respectively) lost the ability to grow on SA (Figure 2B). Additionally, the *vanA2B2*, PSN_2687, and PSN_2688 deletion mutants (Δ*vanA2B2*, Δ2687, and Δ2688, respectively) presented retarded growth on SA compared with the wild-type strain. In contrast, the PSN_2683, PSN_2684, PSN_2685, PSN_2686, PSN_2690, PSN_2697, PSN_2698, and PSN_2699 (*fdhA2*) deletion mutants (Δ2683, Δ2684, Δ2685, Δ2686, Δ2690, Δ2697, Δ2698, and Δ2699, respectively) exhibited growth equivalent to that of the wild-type strain (Figure 2C). To rule out the possibility that the observed phenotypic changes were due to polar effects, we evaluated the growth of complemented strains on SA. The introduction of complementary plasmids into the Δ*vanA1B1*, Δ2687, Δ2688, Δ2689, Δ2693–2694, Δ2700, Δ2701, and Δ2702 mutants restored their ability to grow on SA as the wild-type strain (Figure S2), confirming that these genes are indeed involved in SA catabolism. In contrast, complementation of the Δ*vanA2B2* mutant did not restore its ability to grow on SA, indicating that *vanA2B2* is not involved in SA catabolism.

**Figure 2:**
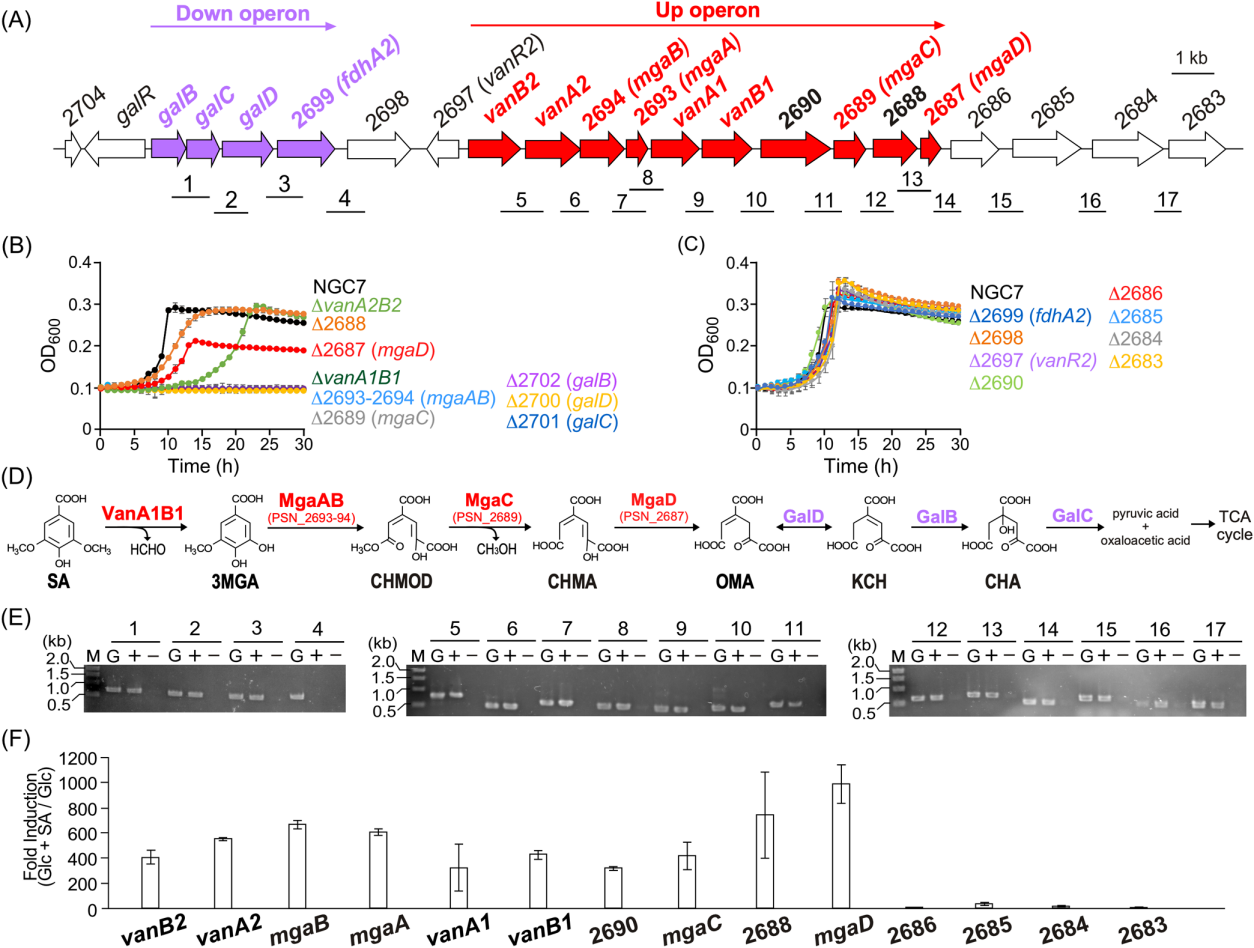
SA catabolic pathway and SA catabolic gene cluster in NGC7. (A) Genetic map around *vanA1B1*. The numbered bars below the map indicate the regions targeted for amplification and correspond to the numbering in panel (E). Red and purple arrows indicate the Up and Down operons, respectively. (B, C) Growth of mutants on SA. Mutants exhibiting growth retardation on SA compared with NGC7 (B) and mutants whose growth was equal to that of NGC7 (C). Growth of NGC7, Δ*vanA2B2*, Δ2688, Δ2687, Δ*vanA1B1*, Δ2693–2694, Δ2689, Δ2702, Δ2700, Δ2701, Δ2699, Δ2698, Δ2697, Δ2690, Δ2686, Δ2685, Δ2684, and Δ2683 cells in 5 mM SA monitored by measuring the OD_600_. (D) Putative SA catabolic pathway. Abbreviations: SA, syringic acid; 3MGA, 3-*O*-methylgallic acid; CHMOD, 4-carboxy-2-hydroxy-6-methoxy-6-oxohexa-2,4-dienoic acid; CHMA, 4-carboxy-2-hydroxymuconic acid; OMA, 4-oxalomesaconic acid; KCH, 2-keto-4-carboxy-3-hexenedioic acid; CHA, 4-carboxy-4-hydroxy-2-oxoadipic acid. Enzymes: VanA1 and VanB1, SA *O*-demethylase oxygenase and oxidoreductase subunit, respectively; MgaA and MgaB; putative 3MGA dioxygenase α and β subunit, respectively; MgaC; putative CHMOD hydrolase; MgaD, putative CHMA tautomerase; GalD, OMA tautomerase; GalB, KCH hydratase; GalC, CHA aldolase. (E) RT-PCR analysis. Lanes: M, molecular size markers; G, control PCR with the NGC7 genomic DNA; + and −, RT-PCR with and without reverse transcriptase, respectively. (F) Fold induction of gene expression from *vanB2* to PSN_2683. The mRNA levels in NGC7 cells grown on 5 mM Glc or 5 mM Glc + 5 mM SA were quantified via qRT-PCR. The fold changes in the mRNA levels of NGC7 cells grown on Glc + SA relative to those grown on Glc are indicated. Each value represents the mean ± standard deviation (error bars) from three independent experiments.

Next, we predicted the function of each gene on the basis of findings from known SA catabolic pathways (Figure 1). In DSM 12444 and SYK-6, 3MGA, produced by the O demethylation of SA, undergoes aromatic ring cleavage to form CHMOD or PDC. CHMOD is subsequently converted to PDC nonenzymatically or by hydrolysis and isomerization to OMA; PDC is then hydrolyzed to OMA^15,30^ (Figure 1). Additionally, in an alternative pathway, 3MGA undergoes O demethylation and conversion to gallic acid, followed by aromatic ring cleavage to OMA.^15,31^ PSN_2693 and PSN_2694 exhibit 47 and 41% amino acid sequence identity to the C-terminus (positions 214 to 331) and N-terminus (positions 1 to 208) of GalA from KT2440 (Figure S3), respectively. They also exhibit 21 and 41% identity to the α (LigA) and β (LigB) subunits of the protocatechuic acid 4,5-dioxygenase from SYK-6 (Figure S4). These findings suggest that the products of PSN_2693 and PSN_2694 form a heterodimeric aromatic ring cleavage dioxygenase similar to LigAB.^53^ Therefore, we designated PSN_2693 and PSN_2694 as *mgaA* and *mgaB*, respectively (Figure 2D). Upon aromatic ring cleavage by dioxygenase, 3MGA is converted to CHMOD or PDC^16,30,31^ (Figure 1). No genes were found in NGC7 that share more than 30% amino acid sequence identity with DesC, DesD, and LigI, which are involved in the conversion of CHMOD or PDC in DSM 12444. On the other hand, no significant similarity was found between PSN_2689 and DesC (13% identity), but both possess a MenH domain (COG0596) and are classified as α/β-fold hydrolases (Figure S5). If PSN_2689 is responsible for CHMOD hydrolysis, it is predicted that CHMOD accumulated is nonenzymatically converted to PDC during SA catabolism in the Δ2689 mutant (Figure 1). To evaluate this hypothesis, we analyzed the products generated when Δ2689 grew in the presence of SA. While PDC did not accumulate in the wild-type strain, Δ2689 produced PDC in an equivalent amount to the added SA after 12 h of cultivation (95% mol/mol), and PDC remained after 24 h (Figure S6). These results strongly suggest that PSN_2689 hydrolyzes CHMOD into 4-carboxy-2-hydroxymuconic acid (CHMA) in NGC7; thus, PSN_2689 was designated *mgaC*. Furthermore, the analysis of Δ*mgaC* indicated that NGC7 cannot convert PDC. CHMA is predicted to be converted to OMA by a tautomerase.^15^ PSN_2687 has a SnoaL-like domain (pfam13577) and is predicted to belong to the nuclear transport factor 2 family, which includes isomerases (Figure S7A). Thus, we predicted that PSN_2687 catalyzes the isomerization of CHMA and designated it *mgaD*. PSN_2700, PSN_2702, and PSN_2701 share 62, 68, and 82% amino acid sequence identity with GalD, GalB, and GalC, respectively, which are involved in gallic acid catabolism in KT2440 ^12,40^ (Figure 1). Thus, we designated PSN_2700, PSN_2702, and PSN_2701 as *galD*, *galB*, and *galC*, respectively. PSN_2688 was predicted to be a transporter because of the presence of a Phenol_MetA_deg domain (pfam13557) found in the outer membrane channels (Figure S7B). On the basis of all the above findings, we deduced the SA catabolic pathway of NGC7, as shown in Figure 2D. SA is O demethylated to 3MGA by VanA1B1, subsequently converted to OMA by MgaAB, MgaC, and MgaD, and further catabolized to pyruvic acid and oxaloacetic acid by GalD, GalB, and GalC, eventually entering the TCA cycle.

### Operon Structure of the SA Catabolism Genes Cluster

Among the genes induced in the presence of SA that share the same transcriptional orientation, we focused on *galB*-PSN_2698 and *vanB2*-PSN_2683 and performed RT-PCR analysis. Amplification products were observed in the *galB-fdhA2* and *vanB2*-PSN_2683 regions (Figure 2A,E). As shown by RNA-seq analysis, all of the genes from *galB* to *fdhA2* were significantly induced (94- to 250-fold) in the presence of SA (Table S4), suggesting that these genes constitute an operon. In contrast, the induction levels from *vanB2* to PSN_2683 markedly decreased between *mgaD* and PSN_2686 (Table S4), suggesting that transcription terminates in the intergenic region between *mgaD* and PSN_2686. Therefore, qRT-PCR analyses were performed. The transcription of genes from *vanB2* to *mgaD* was induced 320- to 990-fold in the presence of SA, whereas the induction levels of genes downstream of *mgaD* decreased by 5.4- to 41-fold, supporting the RNA-seq data (Figure 2F). On the basis of these results, we infer that the induction observed from PSN_2686 to 2683 resulted from a read-through that originated from *vanB2*; thus, we concluded that *vanB2* to *mgaD* constitute an operon. The *vanB2-mgaD* operon and the *galB-fdhA2* operon include genes encoding enzymes involved in the conversion of SA to OMA and OMA to pyruvic acid and oxaloacetic acid, respectively. Therefore, these operons were designated the SA catabolic upstream and downstream pathway operons (Up and Down operons) (Figure 2A).

### Transcription of the Up Operon is Regulated by the VanR2 Repressor

We focused on the putative GntR family transcriptional regulators encoded by PSN_2697 and PSN_3756 (*vanR*) as candidate regulators of the Up operon. PSN_2697 is located directly upstream of the Up operon in a reverse transcriptional orientation (Figure 2A), whereas *vanR* is located directly downstream of *vanA4B4*, which encodes the protein responsible for VA O demethylation and shares 92% amino acid sequence identity with KT2440 VanR. PSN_2697 shares 30–33% amino acid sequence identity with VanR from NGC7, KT2440, ATCC 17978,^42^ ADP1,^43^ and NA1000.^23^ To determine whether PSN_2697 and VanR are involved in the transcriptional regulation of the Up operon, qRT-PCR analyses of *vanA1* were performed using the deletion mutants of PSN_2697 (Δ2697) and *vanR* (Δ*vanR*) (Figure 3). The transcription patterns of *vanA1* in the Δ*vanR* mutant were consistent with those in the wild-type strain. In contrast, the transcription levels of *vanA1* in Δ2697 under noninducing conditions increased to the same level as those upon SA induction. These results strongly suggest that the transcription of the Up operon is negatively regulated by PSN_2697. Therefore, we designated PSN_2697 *vanR2*.

**Figure 3:**
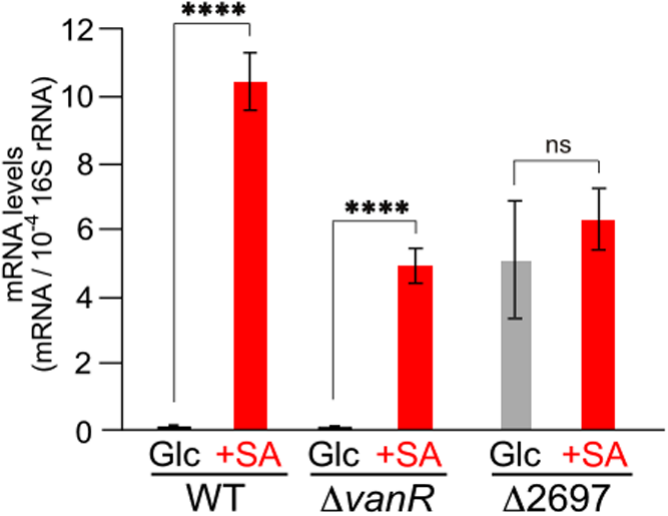
Identification of the transcriptional regulator of the Up operon. qRT-PCR analysis was performed to measure *vanA1* mRNA levels in NGC7, Δ*vanR*, and Δ2697 grown on 5 mM Glc or 5 mM Glc + 5 mM SA. The mRNA levels were normalized to those of 16S rRNA. Each value represents the mean ± standard deviation (error bars) from three independent experiments. Asterisks show statistically significant differences between cells grown in Glc and Glc + SA (ns, *p* > 0.05; ****, *p* < 0.0001).

### Identification of the Promoter Region of the Up Operon and the Binding Site of VanR2

To identify the promoter region of the Up operon, the promoter assay plasmids pB2-1, pB2-2, and pB2-3 were constructed by cloning the DNA fragments containing the stepwise upstream regions of the *vanB2* start codon into the upstream of the *mNeonGreen* gene in the promoter analysis vector pSEVAmNeon (Figures 4A and S8A). Because pSEVAmNeon uses kanamycin resistance as a marker, each plasmid was introduced into Δ*aph*, a kanamycin resistance gene mutant of NGC7, and promoter assays were performed using mNeonGreen-derived fluorescence as an indicator. pB2-1 and pB2-2 exhibited increased promoter activity in the presence of SA, whereas pB2-3 presented activity comparable to that of the vector control (Figures 4A and S8A). In the nonoverlapping region of pB2-2 and pB2-3, we found putative −35 (5′-TTGACT-3′) and −10 (5′-TAACAT-3′) sequences similar to the consensus promoter sequence of (−35, 5′-TTGACC-3′; −10, 5′-TATAAT-3′)^54^ (Figures 4A and S8A). Promoter activity was abolished in pB2-2_-35m and pB2-2_-10m with mutations in the putative −35 and −10 sequences of pB2-2, strongly suggesting that these sequences function as −35 and −10 promoter elements (Figures 4A and S8A).

**Figure 4:**
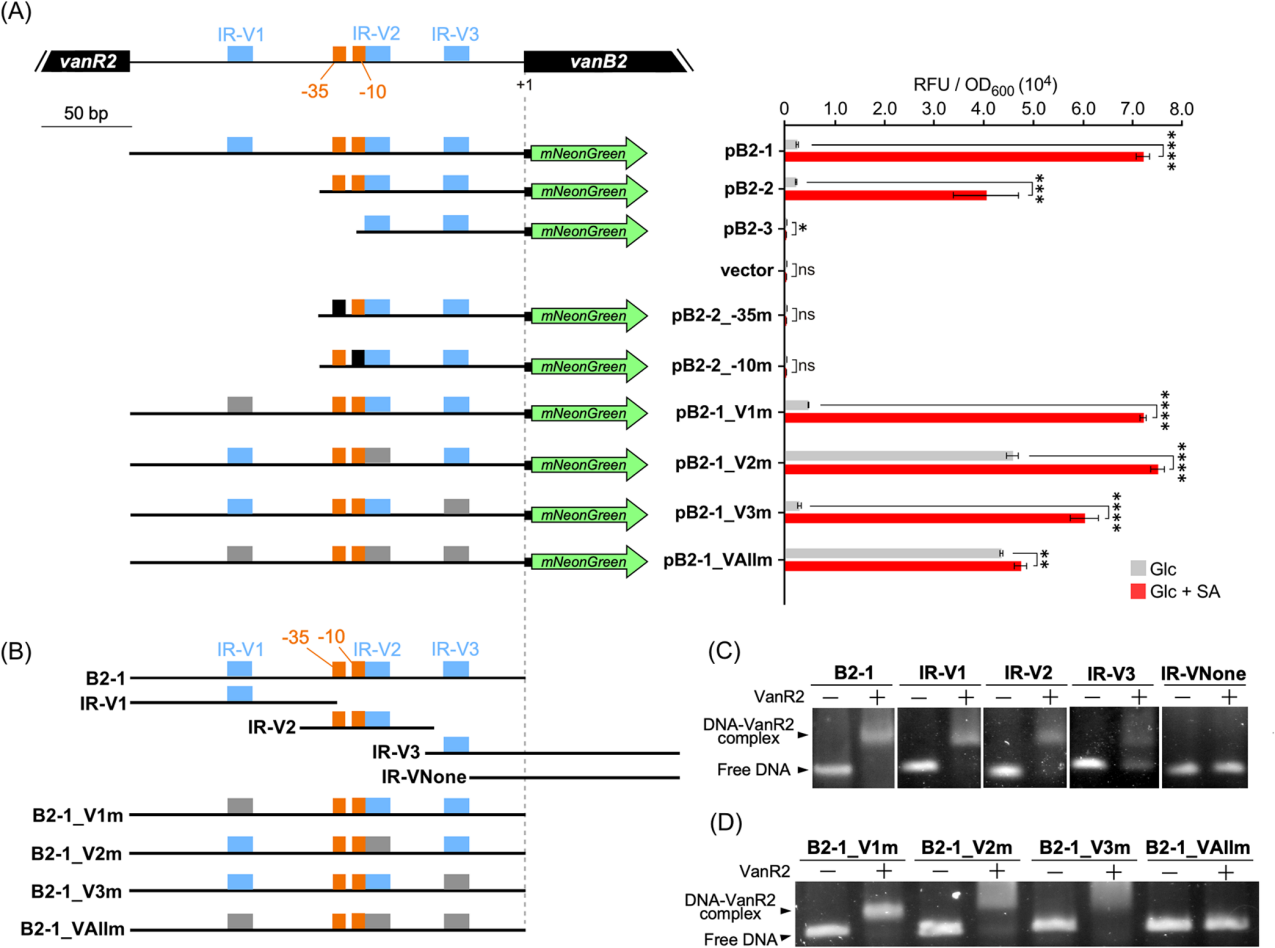
Promoter analysis of the Up operon and VanR2 DNA binding analysis. (A) Promoter analysis. The *vanB2* upstream regions used for the promoter analysis and promoter activities are shown on the left and right, respectively. Target regions were inserted upstream of *mNeonGreen* in the pSEVAmNeon vector to construct reporter plasmids. Because pSEVAmNeon uses kanamycin resistance as a marker, the kanamycin resistance gene mutant Δ*aph*, was used as the wild-type control for promoter assays. Δ*aph* cells harboring each reporter plasmid were grown on 5 mM Glc or 5 mM Glc + 5 mM SA. Promoter activity is shown as relative fluorescence units (RFU) of mNeonGreen normalized to the OD_600_. The DNA fragments are shown by black bars. The following colored boxes indicate each motif: IR motif (5′-GGGATCCC-3′, light blue), mutated IR motif (5′-AAAGTCCC-3′, gray), promoter sequence (−35, 5′-TTGACT- 3′ and −10, 5′-TAACAT-3′, orange), and mutated promoter sequence (−35, 5′-CCAGTC-3′ and −10, 5′-CGGTGC-3′, black). Each value represents the mean ± standard deviation (error bars) from three independent experiments. Asterisks show statistically significant differences between cells grown in Glc and Glc + SA (ns, *p* > 0.05; *, *p* < 0.1; **, *p* < 0.01; ***, *p* < 0.001; ****, *p* < 0.0001). (B) DNA fragments used for EMSAs. (C, D) EMSAs of the binding of VanR2 using fragments with IR deletions (C) and IR mutations (D). The gel images show separation of the binding reaction mixture on 2% agarose.

For electrophoretic mobility shift assays (EMSAs), *vanR2* was expressed in as a C terminal His tag-fused protein and purified by Co^2+^ affinity chromatography. Sodium dodecyl sulfate-polyacrylamide gel electrophoresis (SDS-PAGE) analysis showed that His-tag-fused VanR2 was purified to near homogeneity (Figure S9). EMSAs were performed to determine the DNA binding sites of VanR2 (Figure 4B,C). When the B2-1 probe, containing the full-length *vanR2-vanB2* intergenic region, was used, a shifted band attributed to the DNA−VanR2 complex was observed. This result indicates that VanR2 binds to the promoter region of the Up operon. Upon searching for repeat sequences to which VanR2 may bind in the *vanR2-vanB2* intergenic region, three identical inverted repeats (IRs, 5′-GGGATCCC-3′), IR-V1, IR-V2, and IR-V3, were found (Figure 4B). Shifted bands were observed for the IR-V1, IR-V2, and IR-V3 probes (containing only one of each IR) but not for the IR-VNone probe (containing no IR) (Figure 4C). Furthermore, the shifted band intensity of the IR-V3 probe was weaker than those of the IR-V1 and IR-V2 probes, and more free DNA was observed. EMSAs were performed using the B2-1_V1m, B2-1_V2m, B2-1_V3m, and B2-1_VAllm probes containing mutations in IR-V1, IR-V2, IR-V3, and all IRs of the B2-1 probe, respectively. No shifted band was detected only in the B2-1_VAllm probe (Figure 4D). These results indicate that VanR2 binds independently to each IR.

To investigate the role of each IR in transcriptional regulation, we constructed the pB2-1_V1m, pB2-1_V2m, and pB2-1_V3m plasmids with mutations in each IR of pB2-1 and the pB2-1_VAllm plasmid with mutations in all IRs and performed promoter assays (Figure 4A). Mutations in IR-V1 and IR-V3 did not affect promoter activity. In contrast, pB2-1_V2m and pB2-1_VAllm containing the IR-V2 mutation presented increased promoter activity under noninducing conditions, similar to the activity observed upon SA induction. These results indicate that VanR2 binding to IR-V2 is crucial for transcriptional repression of the Up operon.

### SA and 3MGA Are Inducers of the Up Operon

To examine whether SA, 3MGA, and VA are inducers of the Up operon, we constructed a deletion mutant of four *vanAB* homologues and *mgaAB* (Δ*vanA2B2 mgaAB vanA1B1 vanA4B4 vanA3B3*), which lost the ability to convert these compounds (Figures S1 and S10). We subsequently cultured the Δ*vanA2B2 mgaAB vanA1B1 vanA4B4 vanA3B3* mutant cells harboring pB2-1 in the presence of SA, 3MGA, or VA and performed promoter assays. The promoter activity increased 5.9- and 1.9-fold in the presence of SA and 3MGA, respectively, compared with that in the presence of Glc, whereas the promoter activity in the presence of VA was comparable to that in the presence of Glc (Figure 5A). These results indicate that SA and 3MGA act as inducers of the Up operon. Next, we evaluated the effects of both compounds as effectors on the binding of VanR2 to DNA (Figure 5B,C). In the presence of 50 mM SA or 30 mM or more 3MGA, a decrease in band shift and an increase in the free DNA content were observed, suggesting that SA and 3MGA interfere with VanR2 binding to its target DNA. Although these effector concentrations appear high, similarly high concentrations required for DNA-binding dissociation have been reported in EMSA studies of the GntR-family transcriptional regulator PdhR.^55^

**Figure 5:**
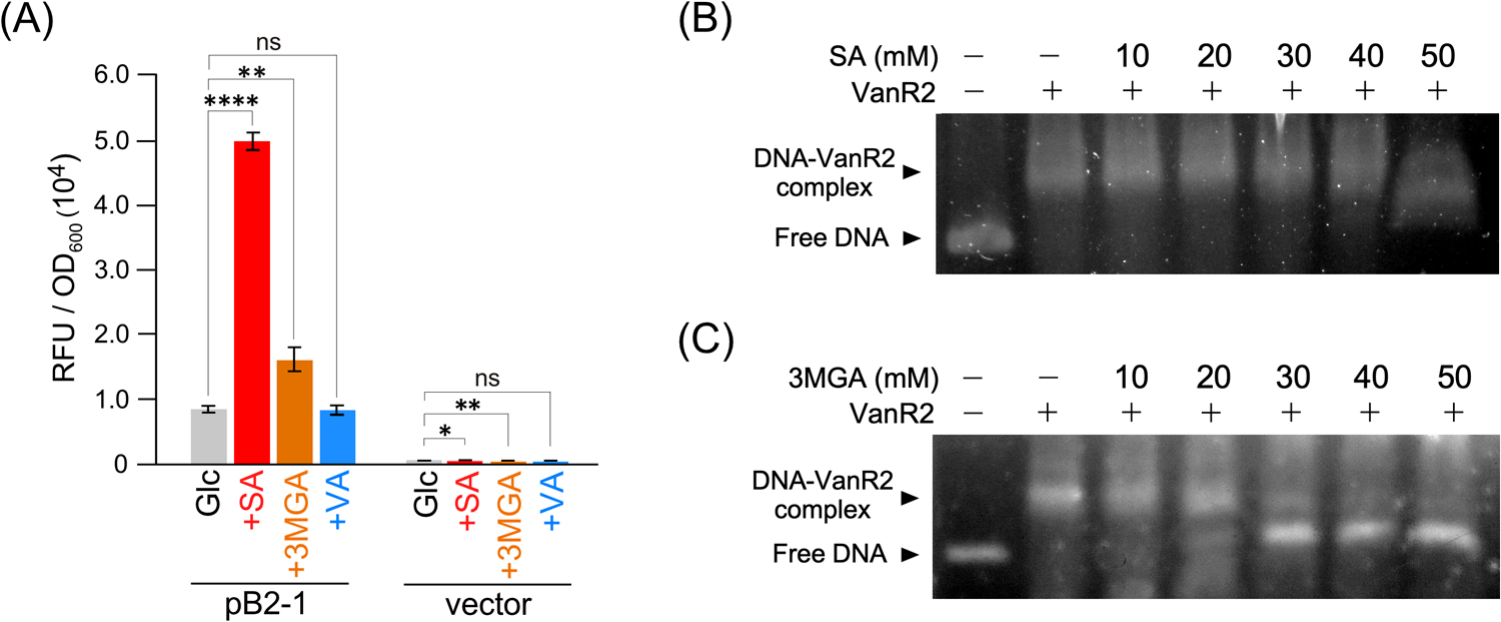
Identification of inducers of the Up operon. (A) Promoter analysis using the SA and 3MGA accumulation mutant. Promoter activities (RFU/OD_600_) of the Δ*vanA1B1 vanA2B2 mgaAB vanA4B4 vanA3B3* mutant cells harboring pB2-1 or the pSEVAmNeon vector grown on 5 mM Glc, 5 mM Glc + 5 mM SA, 5 mM Glc +5 mM 3MGA, and 5 mM Glc + 5 mM VA. Each value represents the mean ± standard deviation (error bars) from three independent experiments. Asterisks show statistically significant differences between the values linked by brackets (ns, *p* > 0.05; *, *p* < 0.05; **, *p* < 0.01; ****, *p* < 0.0001). (B and C) Identification of the effector molecules of VanR2. Purified VanR2 (1.5 pmol/μL) and the B2-1 fragment (8 fmol/μL) were incubated in the presence (10, 20, 30, 40, and 50 mM) or absence of SA (B) or 3MGA (C). The gel images show separation of the binding reaction mixture on 2% agarose.

### VanR2 Represses Its Own Transcription

Since *vanR2* is located directly upstream of *vanB2* in the reverse transcriptional orientation, the binding of VanR2 to the *vanR2-vanB2* intergenic region was expected to affect *vanR2* transcription. The transcriptional induction of *vanR2* was thus evaluated and was found to be induced 6.5-fold in the presence of SA (Figure S11A). Promoter assays targeting the upstream region of *vanR2* revealed that the promoter activity of Δ*vanR2* increased in the absence of SA to a level comparable to that observed in the presence of SA (Figures S8B and S11B). These results indicate that VanR2 negatively regulates its own transcription and that this repression is derepressed in the presence of SA.

### Transcription of the Down Operon is Regulated by the GalR Activator

To investigate the transcriptional induction of the Down operon, we performed qRT-PCR analysis of *galB*, the gene furthest upstream of the Down operon. *galB* was significantly induced (390-fold) during SA culture (Figure 6A), a level comparable to that of genes in the Up operon, indicating that transcription of the Down operon is induced during SA catabolism. *galR* (PSN_2703), located directly upstream of *galB*, shares 49% amino acid sequence identity with the LysR-type transcriptional regulator GalR,^12^ which activates gallic acid catabolism genes in KT2440. GalR from KT2440 has been predicted to recognize and bind to the recognition binding site (RBS; 5′-ATAAANAAANNGNAT-3′) and the activation binding site (ABS; 5′-TNNAANNNNANNCNN-3′).^41^ We found an RBS-like sequence (5′- ATAAGTAAAAAGTAT-3′) upstream of *galB*, while no clear ABS motif was found (Figure S12). We then constructed a deletion mutant of *galR* (Δ*galR*) (Figure S1) and measured the *galB* transcription levels in this mutant. Compared with that in the wild-type strain, the transcription of *galB* in Δ*galR* cultured in the presence of SA was significantly lower (Figure 6A). OMA, an aromatic ring cleavage product of gallic acid, is reported to be an effector molecule of KT2440 GalR.^41^ To investigate the inducer of the Down operon, we used mutants of *mgaC*, *mgaD*, and *galD* (Δ*mgaC*, Δ*mgaD*, and Δ*galD*) involved in CHMOD catabolism as host strains for promoter analysis of the Down operon. The induction of promoter activity observed in Δ*aph* during SA culture was not observed in Δ*mgaC* (Figure 6B). In contrast, Δ*mgaD* and Δ*galD* resulted in 1.5- and 15-fold greater inducibility than Δ*aph*, respectively. These results suggest that OMA and CHMA are strong and weak inducers of the Down operon, respectively.

**Figure 6:**
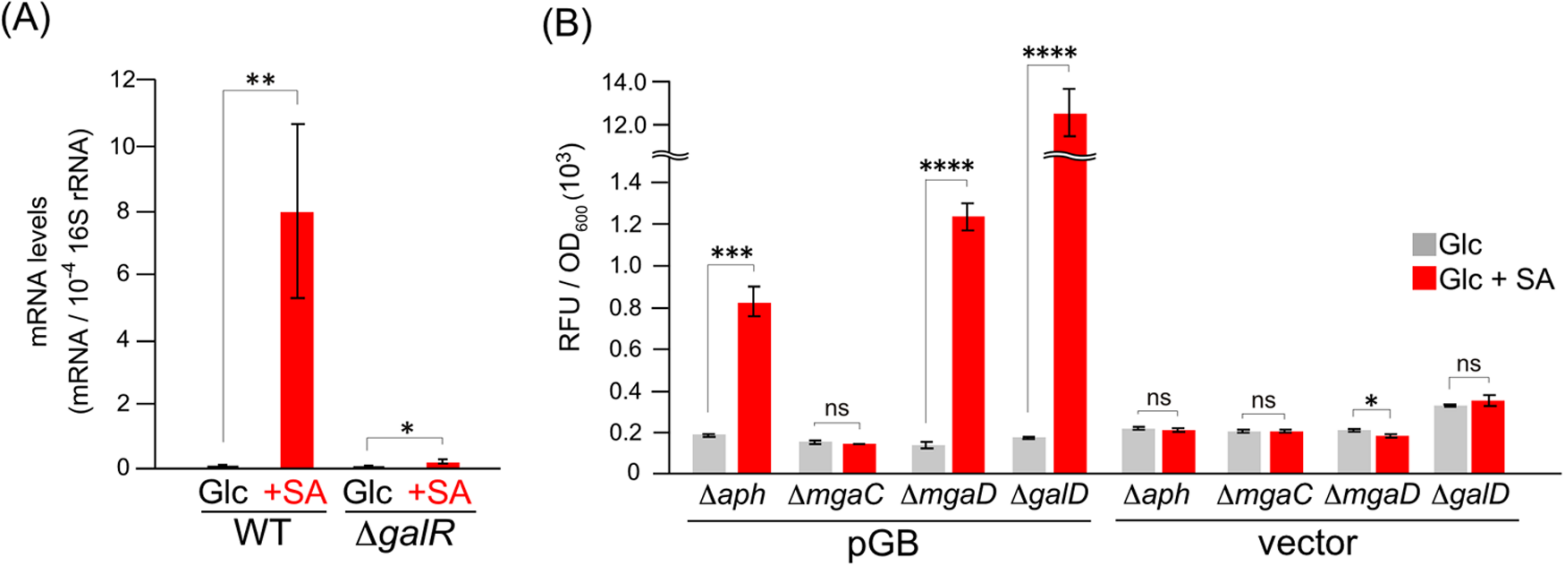
Identification of the transcriptional regulators and inducers of the Down operon. (A) qRT-PCR analysis of *galB*. The mRNA levels of *galB* in NGC7 and Δ*galR* grown on 5 mM Glc and 5 mM Glc + 5 mM SA were quantified via qRT-PCR. The mRNA levels were normalized to those of 16S rRNA. (B) Promoter analysis for inducer identification. Promoter activities (RFU/OD_600_) of Δ*aph* (the wild-type control), Δ*mgaC*, Δ*mgaD*, and Δ*galD* cells harboring pGB, a reporter plasmid containing the upstream region of *galB* or the pSEVAmNeon vector grown on 5 mM Glc or 5 mM Glc + 5 mM SA. Each value represents the mean ± standard deviation (error bars) from three independent experiments. Asterisks show statistically significant differences between cells grown on Glc and Glc + SA (ns, *p* > 0.05; *, *p* < 0.05; **, *p* < 0.01; ***, *p* < 0.001; ****, *p* < 0.0001).

### FdhA2 is Involved in HCHO Detoxification during SA Catabolism

The O demethylation of VA and SA by VanAB and the hydrolysis of CHMOD produce HCHO^24^ and methanol^34^ as byproducts, respectively (Figure 2D). In KT2440, methanol is oxidized to HCHO by PedE, PedH, AdhP, and YiaY.^56^ Therefore, 2 mol of HCHO is estimated to be produced from 1 mol of SA in the SA catabolic pathway of NGC7. In KT2440, HCHO is detoxified to formic acid (HCOOH) via two pathways: the glutathione-dependent pathway involving *S*-(hydroxymethyl)­glutathione dehydrogenase/hydrolase (FrmA/FrmC) and the thiol-independent pathway involving HCHO dehydrogenases (FdhA, FdhB, and AldB-II)^56,57^ (Figure S13). Finally, HCOOH is further oxidized to CO_2_ by HCOOH dehydrogenases (PP_0256-FdhD, FdoGHI-FdhE, FmdEFGH, and PP_4596).^56^ HCHO is generally known to be toxic to bacteria,^58^ and growth inhibition in the presence of HCHO was indeed observed in NGC7 (Figure S14). Therefore, the rapid detoxification of HCHO is crucial for efficient SA catabolism. NGC7 harbors genes encoding enzymes showing more than 88% amino acid sequence identity to the methanol dehydrogenases (*pedE*, *pedH*, *adhP*, and *yiaY*), HCHO dehydrogenases (*fdhA, frmAC, aldB-II*, and *fdhB*), and HCOOH dehydrogenases (PSN_0320*-fdhD, fdoGHI-fdhE, fmdEFGH*, and PSN_4723) in KT2440 (Table S5).^56,57,59^ In addition, NGC7 contains *fdhA2*, which is part of the Down operon, shares 98% amino acid sequence identity with FdhA from KT2440, in addition to *fdhA* in the sarcosine catabolism gene cluster as in KT2440 (Figure 2A and Table S5). RNA-seq analysis showed that multiple genes encoding methanol dehydrogenases (*pedE*, *pedH*, and *yiaY*), HCHO dehydrogenases (*fdhA*, *fdhA2*, *frmAC*, *aldB-II*, and *fdhB*), and HCOOH dehydrogenases (PSN_0320-*fdhD*, *fdoGHI*-*fdhE*, and *fmdEFGH*) were induced 2- to 212-fold during SA cultivation (Table S5). Among these, *fdhA2* showed the highest induction, suggesting its involvement in HCHO detoxification. However, as shown in Figures 2C and 7A, deletion of *fdhA2* did not affect the growth ability of NGC7 on SA. Among the HCHO detoxification enzymes, FrmAC has major role in HCHO detoxification in KT2440.^56^ Therefore, we constructed an *frmAC* mutant (Δ*frmAC*) and an *fdhA2* and *frmAC* double mutant (Δ*fdhA2 frmAC*) (Figure S1). The growth of Δ*frmAC* on SA was delayed, whereas Δ*fdhA2 frmAC* was unable to grow on SA (Figure 7A). qRT-PCR analysis revealed that *frmA* is constitutively expressed, whereas *fdhA2* was induced 390-fold when cultured with SA compared with culture with Glc (Figure 7B). Furthermore, *fdhA2* transcription was 21-fold higher than that of *frmA* in the presence of SA. These results show that the inducible *fdhA2* and the constitutively expressed *frmAC* are involved in HCHO detoxification during SA catabolism.

**Figure 7:**
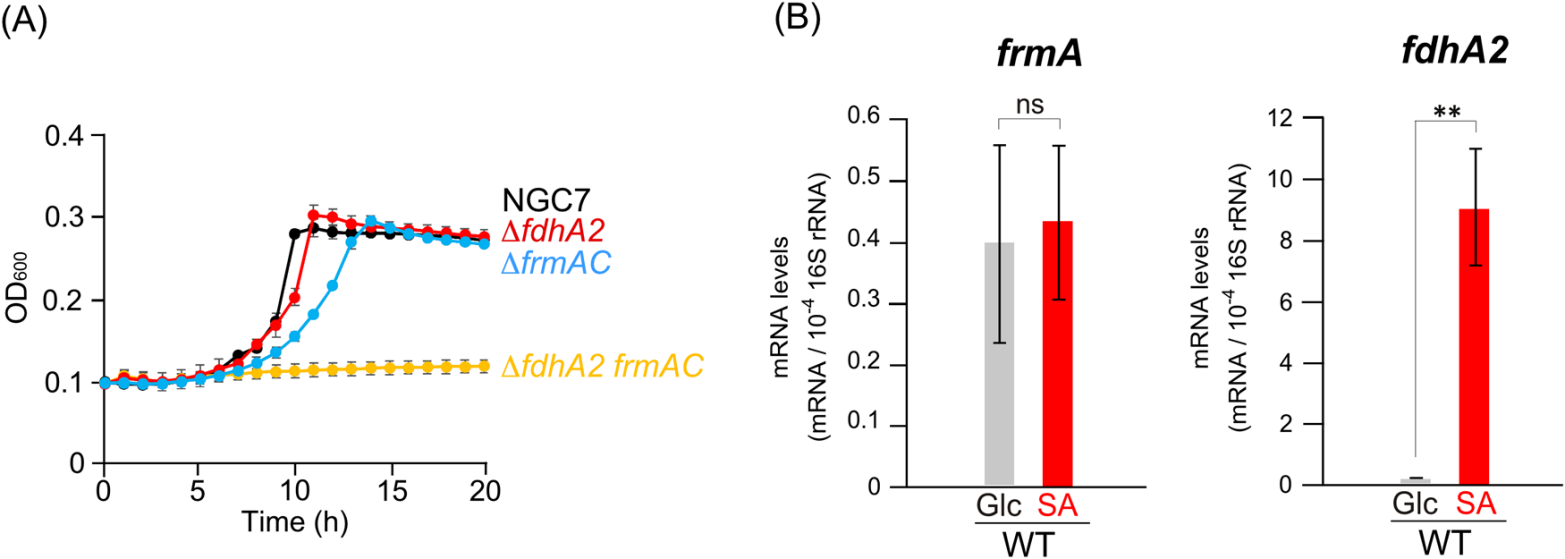
Role of *fdhA2* and *frmAC* in HCHO detoxification. (A) Growth of the *fdhA2* and *frmAC* mutants on SA. The growth of NGC7, Δ*fdhA2*, Δ*frmAC*, and Δ*fdhA2 frmAC* on 5 mM SA was monitored by measuring the OD_600_. (B) qRT-PCR analysis of *frmA* and *fdhA2*. The mRNA levels of *frmA* and *fdhA2* in NGC7 grown on 5 mM Glc or 5 mM SA were quantified via qRT-PCR The mRNA levels were normalized to those of 16S rRNA. Each value represents the mean ± standard deviation (error bars) from three independent experiments. Asterisks show statistically significant differences between cells grown in Glc and SA (ns, *p* > 0.05; **, *p* < 0.01).

## Discussion

Bacterial SA catabolic pathways have been clarified in SYK-6 and DSM 12444. In both strains, SA undergoes O demethylation and is converted to 3MGA. As shown in Figure 1, the 3MGA catabolic pathway branches into three distinct pathways. The following discussion focuses on the 3MGA catabolism in NGC7. (i) NGC7 lost its ability to grow on SA when *mgaAB* was deleted, indicating that 3MGA is catabolized via aromatic ring cleavage without undergoing O demethylation to form gallic acid (Figure 2B). (ii) No PDC hydrolase gene was found in NGC7, and Δ*mgaC* accumulated PDC during SA catabolism, indicating that NGC7 cannot convert PDC (Figure S6). In addition, no PDC accumulation was observed in the wild-type strain, suggesting that the aromatic ring cleavage of 3MGA by MgaAB produces only CHMOD (Figure S6). (iii) CHMOD has an estimated half-life of approximately 70 min and is spontaneously converted to PDC.^35^ It is thus deduced that CHMOD produced by the aromatic ring cleavage of 3MGA is not nonenzymatically cyclized to PDC but instead rapidly hydrolyzed to CHMA by MgaC. On the basis of these findings, we concluded that 3MGA is catabolized in NGC7 through a single linear pathway involving aromatic ring cleavage by MgaAB and hydrolysis of CHMOD by MgaC, which differs from the previously reported branched pathways (Figures 1 and 8).

**Figure 8:**
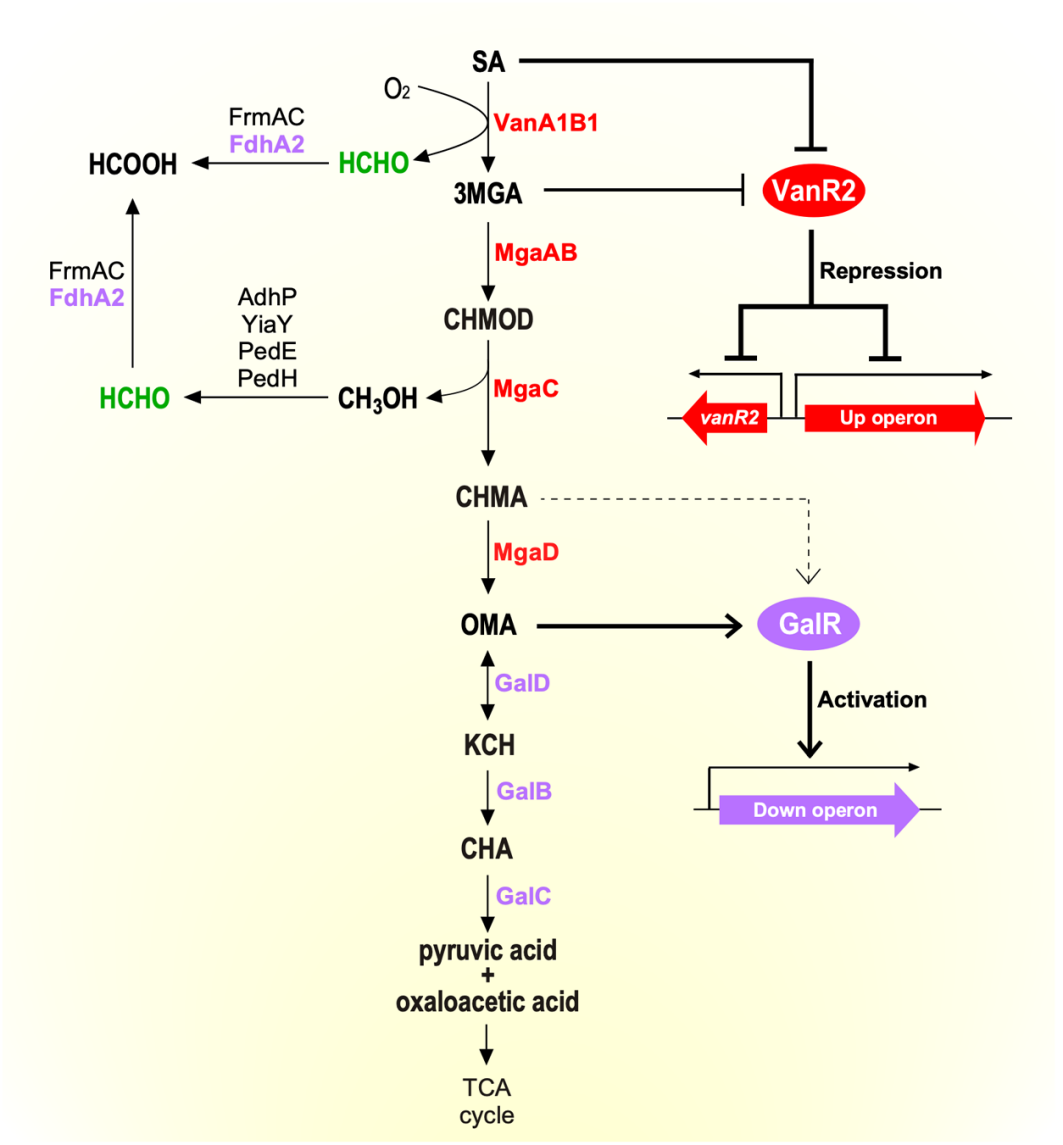
SA catabolic system in *Pseudomonas* sp. NGC7. The SA catabolism pathways, along with the conversion of its byproducts HCHO and methanol, are shown. An overview of the transcriptional regulatory systems involving VanR2 and GalR is shown on the right. Enzymes and genes/operons regulated by VanR2 and GalR are colored red and purple, respectively. Abbreviations: SA, syringic acid; 3MGA, 3-*O*-methylgallic acid; CHMOD, 4-carboxy-2-hydroxy-6-methoxy-6-oxohexa-2,4-dienoic acid; CHMA, 4-carboxy-2-hydroxymuconic acid; OMA, 4-oxalomesaconic acid; KCH, 2-keto-4-carboxy-3-hexenedioic acid; CHA, 4-carboxy-4-hydroxy-2-oxoadipic acid; HCHO, formaldehyde; HCOOH; formic acid. Enzymes: VanA1 and VanB1, SA *O*-demethylase oxygenase and oxidoreductase subunit, respectively; MgaA and MgaB; putative 3MGA dioxygenase α and β subunit, respectively; MgaC; putative CHMOD hydrolase; MgaD, putative CHMA tautomerase; GalD, OMA tautomerase; GalB, KCH hydratase; GalC, CHA aldolase; AdhP, alcohol dehydrogenase zinc-containing; YiaY, alcohol dehydrogenase iron-containing. PedE, pyrroloquinoline quinone (PQQ)-dependent alcohol dehydrogenase; PedH, PQQ-dependent alcohol dehydrogenase; FrmA, *S*-(hydroxymethyl)­glutathione dehydrogenase; FrmC, *S*-(hydroxymethyl)­glutathione hydrolase; FdhA2, glutathione-independent HCHO dehydrogenase.

The SA catabolic system in NGC7 is summarized based on the findings of catabolic pathways, HCHO detoxification, and transcriptional regulation elucidated in this study (Figure 8). SA is O demethylated to 3MGA by VanA1B1, producing HCHO as a byproduct from the methyl moiety of the methoxy group. 3MGA subsequently undergoes aromatic ring cleavage by MgaAB, generating CHMOD, which is then hydrolyzed by MgaC to CHMA, releasing methanol as a byproduct from the methyl group. CHMA is isomerized to form OMA by MgaD, and finally, OMA is converted to pyruvic acid and oxaloacetic acid by GalD, GalB, and GalC, which then enter the TCA cycle. The methanol released during the SA catabolism is expected to be converted to HCHO by methanol dehydrogenases as in KT2440.^56,59^ HCHO undergoes detoxification to HCOOH by the HCHO dehydrogenases encoded by *frmAC* and *fdhA2* and is eventually converted to CO_2_ by HCOOH dehydrogenases, as in KT2440. This interpretation regarding the metabolism of these byproducts is supported by RNA-seq analysis, which showed that several related genes including *frmAC* and *fdhA2* were induced during SA cultivation (Figure 7B and Table S5). *vanA1B1, mgaAB, mgaC*, and *mgaD*, which are responsible for the upstream SA catabolic pathway, constitute the Up operon. Transcription of the Up operon is negatively regulated by VanR2, and this repression by VanR2 is relieved in the presence of SA or 3MGA. VanR2 also negatively regulates its own gene expression. In contrast, the Down operon, which includes *galBCD* and *fdhA2*, which are downstream of the SA catabolic pathway, is positively regulated by GalR. OMA and CHMA are suggested to act as strong and weak inducers of the Down operon, respectively.

CHMOD produced by aromatic ring cleavage of 3MGA has been reported to be a mixture of stereoisomers.^16,30^ Δ*mgaC* lost the ability to grow on SA (Figure 2B), suggesting that MgaC converts both stereoisomers of CHMOD and produces CHMA as a mixture of stereoisomers. In contrast, Δ*mgaD* exhibited a distinct growth phenotype on SA, reaching a maximum OD_600_ of approximately half that of the wild-type strain. On the basis of these observations, it is hypothesized that MgaD isomerizes one of the stereoisomers of CHMA. Similarly, DesC and DesD, which are involved in SA catabolism in DSM 12444, are predicted to catalyze the same reactions.^15^ CHMA was suggested to act as a weak inducer of the Down operon (Figure 6B), however, in Δ*mgaD*, OMA is likely produced from one of the CHMA isomers during incubation in the presence of SA. Further analysis is needed to confirm whether CHMA truly acts as an inducer of the Down operon.

PSN_2688, a component of the Up operon, contains a Phenol_MetA_deg domain found in outer membrane channels (Figure S7B). Transmembrane domain prediction programs indicate that PSN_2688 possesses a signal peptide and a membrane-spanning barrel domain composed of 12 antiparallel β-strands (Figure S15). The delayed growth of Δ2688 in the presence of SA (Figure 2B) suggests that PSN_2688 is involved in the outer membrane transport of SA or its catabolic intermediates. Additionally, PSN_2690 shares 42% amino acid sequence identity with VanK, which is involved in the inner membrane transport of VA in KT2440.^60^ Although deletion of PSN_2690 did not affect growth on SA (Figure 2C), its colocalization with PSN_2688 in the Up operon suggests that it could participate in the inner membrane transport of SA or its catabolic intermediates incorporated into the periplasmic space by PSN_2688.

Among the DNA-binding sites of VanR2, IR-V1, IR-V2, and IR-V3, the binding of VanR2 to IR-V2 appears to be crucial for the transcriptional repression of the Up operon (Figure 4A). Since IR-V2 is located directly downstream of the −10 sequence, the binding of VanR2 to IR-V2 would interfere with RNA polymerase progression. A similar repression mechanism has been proposed for the GntR-type transcriptional regulator FadR from , where the binding site of FadR is located downstream of the −10 sequence of *fadB*, which is regulated by FadR.^61,62^ Although IR-V3 is also located downstream of the promoter sequence, mutation in IR-V3 did not affect the promoter activity of *vanB2* (Figure 4A). This is likely due to the lower binding affinity of VanR2 for IR-V3 than for IR-V1 or IR-V2 (Figure 4C). Therefore, VanR2 preferentially binds to IR-V2 and IR-V1 rather than IR-V3 in vivo, and transcriptional repression is unlikely to occur upon binding to IR-V3. In contrast, IR-V1 is located 28 bp upstream of the −35 sequence, and its mutation did not affect promoter activity (Figure 4A). This finding suggests that VanR2 binding to IR-V1 does not repress transcription of the Up operon. In contrast, putative *vanR2* promoter sequences were found to overlap with IR-V1, suggesting that VanR2 binding to IR-V1 may repress its own transcription (Figures S8B and S11B).

HCHO is known to be toxic to bacteria, acting as a strong electrophile that reacts with DNA and proteins.^58^ HCHO produced by the O demethylation of VA by VanAB was shown to be toxic to .^63^ In VA catabolism, 1 mol of HCHO is produced from 1 mol of VA via O demethylation. In contrast, 1 mol of SA ultimately produces 2 mol of HCHO during SA catabolism in NGC7 (Figure 8). Different toxic effects during VA and SA catabolism, corresponding to differences in the amount of HCHO production, have been observed in KT2440. In engineered KT2440, which acquired the ability to grow on SA by overexpressing *vanAB*, more significant toxicity was observed during SA catabolism than during VA catabolism.^26^ KT2440 possesses only one *fdhA* in its genome.^56,57^ In contrast, NGC7 contains *fdhA2* within the Down operon (Figure 2A), whose expression is induced during SA catabolism, in addition to *fdhA*, and appears to play a role in enhancing the HCHO detoxification capacity. *fdhA2* is presumed to be a paralog of *fdhA*, sharing 85% and 96% nucleotide and amino acid sequence identity, respectively. It is inferred that *fdhA* was duplicated and incorporated into the Down operon as *fdhA2*, enabling efficient detoxification of HCHO during SA catabolism. Meanwhile, *fdhA* in NGC7 is located adjacent to genes involved in sarcosine catabolism, a pathway in which HCHO is produced as a byproduct, as is also the case in KT2440 and PAO1.^56,64^ This gene arrangement suggests that *fdhA* primarily functions in HCHO detoxification during sarcosine catabolism.

In recent years, lignin has gained attention as an unutilized lignocellulosic biomass. Microbial funneling technologies have been developed to produce value-added chemicals, such as polymer building blocks, from mixtures of heterogeneous aromatic compounds obtained by the chemical decomposition of lignin. NGC7 catabolizes all H-, G-, and S-type lignin-derived aromatic monomers and is a promising microbial chassis for producing value-added polymer feedstocks from lignocellulosic biomass through biological funneling processes. To date, production systems have been developed for *cis*,*cis*-muconic acid from sugar cane bagasse alkaline extract^49^ and the catalytic oxidation products of rice husk lignin,^51^ as well as for VA from the catalytic oxidation products of sulfite lignin,^50^ have been developed. In this study, the Δ*mgaC* mutant accumulated PDC as a metabolite of SA in nearly 100% molar yield (Figure S6). PDC has attracted attention as a building block for high-performance polymers.^65,66^ Several systems that produce PDC from lignin-derived aromatic compounds or glucose as feedstocks have been developed using PpY1100,^4,67^ KT2440,^26,68^ DSM 12444,^69,70^ ,^71^ ATCC 13032,^72^ and^73^ as hosts. Among these, only DSM 12444 and KT2440 can use S-type lignin-derived aromatic compounds as feedstocks. This study suggest the potential of NGC7 as a host for PDC production from lignocellulosic biomass containing S-type lignin. Further evaluation of the production efficiency, substrate range, and scalability will be required to assess the practical feasibility.

This study reveals for the first time the SA catabolic pathway involving the VanAB-type enzyme VanA1B1 in NGC7, highlighting further variations in the catabolic pathways for S-type lignin-derived aromatic compounds in bacteria. We investigated whether other organisms possess SA catabolic pathways involving VanA1B1. First, we searched for homologues of VanA1B1 (>50 or 40% amino acid sequence identity and >70% query coverage) using NCBI protein BLAST, and homologues of *vanA1* and *vanB1* were found in 1775 and 1897 organisms, respectively (Figure S16). These organisms represent nine classes: Alphaproteobacteria, Betaproteobacteria, Gammaproteobacteria, Actinomycetes, Flavobacteriia, Cytophagia, Terriglobia, Cyanophyceae, and Thermoleophilia. Among these classes, Alphaproteobacteria and Actinomycetes accounted for the majority, whereas Gammaproteobacteria, which includes *Pseudomonas*, were less common. Surprisingly, only eight *Pseudomonas* strains containing both *vanA1* and *vanB1* were found in addition to NGC7 (Figure S17). In addition, the genomic GC content of NGC7 is 62%, whereas the GC content of *vanA1B1* is 52%. This discrepancy, along with the rate distribution of *vanA1B1* in *Pseudomonas* strains, suggests that *vanA1B1* in NGC7 was most likely acquired through horizontal gene transfer from Alphaproteobacteria, Actinomycetes, Betaproteobacteria, or Gammaproteobacteria. The downstream pathways of 3MGA catabolism have been reported in the 3MGA catabolic pathway in NGC7, SYK-6, and DSM 12444;^10,15^ the gallic acid catabolic pathway in KT2440;^12^ and the protocatechuic acid 4,5-cleavage pathway, which produces OMA as an intermediate, in SYK-6, DSM 12444, *Comamonas* sp. E6, and others.^74,75^ We further investigated whether organisms harboring *vanA1B1*-like genes also possess enzyme genes involved in 3MGA catabolism (>50% amino acid sequence identity, > 70% query coverage) (Figure 9 and Table S6) and identified organisms predicted to contain all the enzymes responsible for converting SA into pyruvic acid and oxaloacetic acid in Gammaproteobacteria, Betaproteobacteria, Alphaproteobacteria, and Actinomycetes. Focusing on *Pseudomonas* strains carrying both *vanA1* and *vanB1*, we conducted the same search and found that one strain is predicted to harbor catabolic enzymes similar to those in NGC7 (Figure S17). This finding suggests that S-type lignin-derived aromatic compounds, in addition to the well-reported G-type lignin-derived aromatic compounds, are degraded by a wide range of bacterial taxa.

**Figure 9:**
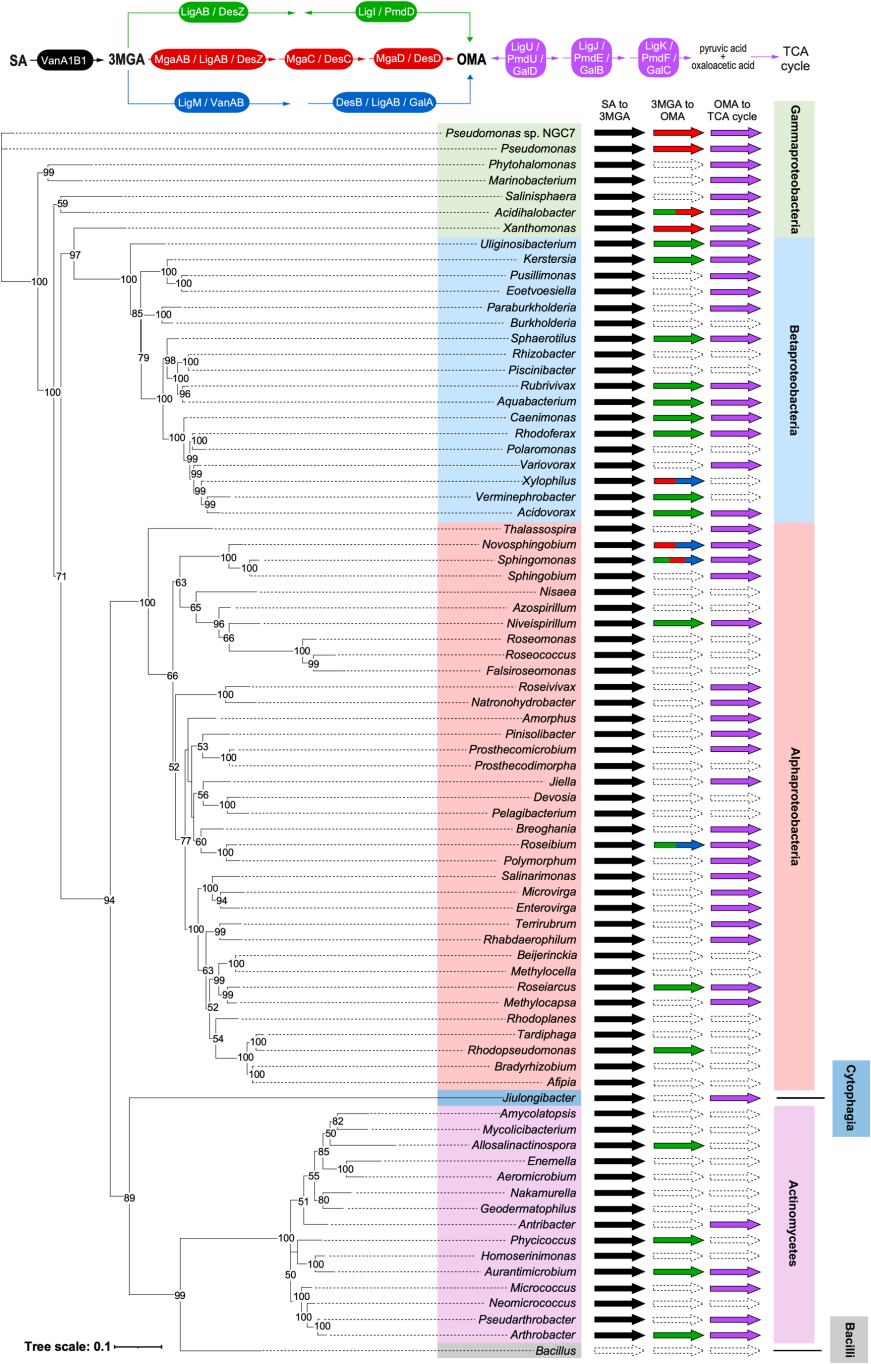
Conservation of SA catabolism genes in bacteria. Among the organisms containing homologues of *vanA1* and *vanB1*, one strain was selected from each genus to examine the conservation of SA catabolism genes (>50% amino acid sequence identity, > 70% query coverage). The enzymes and organisms used in this investigation are listed in Table S6 and Supporting Information Data Sheet 3, respectively. The previously reported SA catabolic pathways are illustrated above. Strains in which both *vanA1* and *vanB1* are responsible for converting SA to 3MGA are indicated by black arrows. Among the branched 3MGA to OMA catabolic pathways, strains containing *ligAB*/*desZ* and *ligI*/*pmdD*, *mgaAB*/*ligAB*/*desZ* and *mgaC*/*desC*, or *ligM*/*vanAB* and *desB*/*ligAB*/*galA* are indicated by green, red, and blue arrows, respectively. Strains with *ligU*/*pmdU*/*galD*, *ligJ*/*pmdE*/*galB*, and *ligK*/*pmdF*/*galC*, which are responsible for converting OMA to products that enter the TCA cycle, are indicated by purple arrows. Thus, strains with three arrows possess one of the enzyme-encoding genes for all reaction steps from SA to the TCA cycle. As *mgaD* and *desD* are not essential for SA catabolism, strains lacking these genes were also counted as having a reaction step from 3MGA to OMA. A phylogenetic tree based on the 16S rRNA of each strain, generated using the maximum likelihood method, is shown on the left. If bootstrap values (based on 1000 replicates) of 50 or more are indicated, they are shown at the branch points. The scale corresponds to 0.1 amino acid substitutions per position. *Bacillus subtilis* subsp. *subtilis* str. 168 (GCF_000155325.1) was used as an outgroup. The class of each strain is marked by colored boxes. Strains possessing none of the SA catabolism genes were excluded from this figure.

## Supplementary Material





## Notes

Bacterial strains, plasmids, primers, culture conditions, and chemicals; genetic manipulation, sequence analysis, phylogenetic analysis, and statistical analysis; construction of deletion mutants and complementation strains; conversion assays for VA, SA, and 3MGA and analysis of SA degradation products; RNA-seq analysis and gene expression profiling; preparation and purification of VanR2 protein; genes in the vicinity of *vanA1B1* and their transcript levels; putative methanol, HCHO, and HCOOH dehydrogenases and their transcript levels; enzymes used to investigate the conservation of SA catabolism genes; growth of complementation strains; amino acid sequences and domain structures of MgaA, MgaB, MgaC, and MgaD; HPLC analysis of SA degradation products; the nucleotide sequence of the intergenic region *vanR2*-*vanB2* and *galR*-*galB*; expression of *vanR2* in and purification; regulatory analysis of *vanR2*; the HCHO detoxification pathways in KT2440; growth inhibition by HCHO; prediction of transmembrane domain and signal peptide; conservation of *vanA1B1* in bacteria; conservation of SA catabolism genes in *Pseudomonas* (PDF)
VanA1 homologues found in bacteria; VanB1 homologues found in bacteria; organisms used for phylogenetic analysis in Figure 9; conservation of SA catabolism genes in *Pseudomonas* (Supporting Information Data Sheets 1–4) (XLSX)
