# Supplementary material for: Catabolic System of Syringic Acid, a Key Intermediate of Lignin-Derived Aromatic Compounds, via a Novel Linear Pathway in Pseudomonas sp. NGC7
Source: J Agric Food Chem. 2025 Jul 16;73(30):18899–913. doi: 10.1021/acs.jafc.5c04544 (PMC12314914; doi:10.1021/acs.jafc.5c04544)
Supplement: Supplementary file 1 [file jf5c04544_si_001.pdf]

Supporting Information

Catabolic System of Syringic Acid, a Key Intermediate of Lignin-Derived Aromatic Compounds, via a Novel Linear Pathway in *Pseudomonas* sp. NGC7

Zen Ookawa,[a] Yudai Higuchi,[b] Masaya Fujita,[a] Tomonori Sonoki,[b] Naofumi Kamimura,[a*] and Eiji Masai,[a]

[a]Department of Materials Science and Bioengineering, Nagaoka University of Technology, Nagaoka, Niigata 940-2188, Japan

[b] Faculty of Agriculture and Life Science, Hirosaki University, Hirosaki, Aomori 036-8561, Japan

*Correspondence:
Naofumi Kamimura
Department of Materials Science and Bioengineering, Nagaoka University of Technology, Nagaoka, Niigata 940-2188, Japan
E-mail: zkami@vos.nagaokaut.ac.jp
TEL: +81 258 47 9402

MATERIALS AND METHODS

Bacterial strains, plasmids, primers, culture conditions, and chemicals

The strains, plasmids, and PCR primers used in this study are listed in Table S1 and S2. *Pseudomonas* sp. NGC7 and its mutants were grown at 30°C with shaking (160 rpm) in lysogeny broth (LB) or MMx-3 medium [1] containing glucose (Glc), SA, VA, HCHO, or 3MGA. When necessary, the media were supplemented with 50 mg/L kanamycin. *Escherichia coli* was grown in LB at 30 or 37°C and used for the cloning and gene expression experiments. The media for *E. coli* transformants were supplemented with 100 mg/L ampicillin or 25 mg/L kanamycin. SA and VA were purchased from the Tokyo Chemical Industry (Japan). 3MGA was purchased from BLD Pharmatech (China). HCHO was purchased from Sigma-Aldrich Japan (Japan). Glc was purchased from FUJIFILM Wako Pure Chemical Corporation (Japan). PDC was prepared as described previously [2].

Genetic manipulation, sequence analysis, phylogenetic analysis, and statistical analysis

Gene cloning was performed using NEBuilder HiFi DNA Assembly Cloning kit (New England Biolabs, USA). DNA sequencing was performed by Eurofins Genomics (Japan). Gene synthesis was performed by Eurofins Genomics or Twist Bioscience (USA). DNA and protein sequences were obtained from NCBI database. Pairwise alignment was carried out using EMBOSS Needle [3]. Multiple alignment was generated by MAFFT [4] and SINA [5]. NLM's conserved domain database [6] was used to search for a domain. Homologous genes were searched using NCBI Protein BLAST (BLAST-P) [7]. Transmembrane and signal peptide prediction was performed by BOCTOPUS2 [8] and SignalP 6.0 [9] programs. Phylogenetic trees were generated based on the maximum likelihood method algorithm using the IQ-TREE2 [10], with branch stability assessed by ultrafast bootstrap approximation (1000 replicates). Phylogenetic trees were visualized in iTOL v6 [11]. Statistical analyses were performed using GraphPad Prism 10 (GraphPad Software, USA). Pairwise comparisons were made using Student's *t*-test with a P value < 0.05 considered statistically significant.

Mutant construction, conversion of VA, SA, and 3MGA, and analysis of SA degradation product

All gene disruption in this paper was performed by deletion of gene coding regions. The PCR primers were designed to amplify ca. 0.5-1.0 kb fragments upstream of the 5' end and downstream of the 3' end of the target gene (Table S2). This design results in either complete deletion or retention 12-153 bp at both the 5′ and 3′ ends after gene deletion (Figure S1). For the simultaneous deletion of multiple

genes, primers were designed to either completely delete the target genes or leave 12-50 bp at the 5′ end of the most upstream gene and at the 3′ end of the most downstream gene of the target region. These plasmids were introduced into cells of kanamycin resistance gene deletion mutant (Δ*aph*) or derivatives of Δ*aph* by electroporation [12]. Electroporation and mutant selection were performed according to a previous report [12]. Gene deletion was confirmed by colony PCR using the primer pairs listed in Table S2. The plasmids used for the gene complementation experiments were constructed by introducing the target gene downstream of the P_{lacIq} promoter in pSEVA2321.

NGC7, NGC751, and NGC753 were cultured in MMx-3 containing 5 mM Glc + 5 mM SA, 5 mM Glc + 5 mM VA, or 5 mM Glc + 5 mM 3MGA. Supernatants of cultures were collected continuously, diluted with 0.1% formic acid, and filtered through a 0.20 μm PTFE membrane (Captiva Econofilter, Agilent). Filtrates were analyzed using high-performance liquid chromatography (HPLC) (ACQUITY UPLC system, Waters) equipped with a TSKgel ODS-140HPT column (particle size: 1.9 μm; dimensions: 2.0 × 100 mm, Tosoh). The mobile phase consisted of a gradient mixture of solution A (acetonitrile containing 0.1% formic acid) and B (water containing 0.1% formic acid) under the following conditions: 0–1.0 min, 3% A; 1.0–2.5 min, linear gradient from 3 to 30% A; 2.5–4.0 min, 0% A; 4.0–4.1 min, linear gradient from 30 to 3% A; 4.1–5.0 min, 3% A. The flow rate was maintained at 0.5 mL/min, and the column temperature was set to 30°C. VA, SA, 3MGA, and PDC were detected at 260 nm, 275 nm, 265 nm, and 315 nm, respectively.

RNA-seq analysis

Cells of NGC7 pre-cultured in LB were collected, washed twice with MMx-3 medium, and resuspended in the same medium. The resultant cell suspensions were inoculated into MMx-3 medium containing 5 mM Glc (non-inducing condition) or 5 mM SA to an optical density at 600 nm (OD_{600}) of 0.2 and incubated to mid-log phase. Total RNA was isolated from the cells using an Illustra RNAspin Mini RNA Isolation Kit (GE Healthcare, Buckinghamshire, UK). The RNA samples were treated with RNase-free DNase I (Takara Bio, Japan), purified by Monarch RNA Cleanup Kit (New England Biolabs), and finally dissolved in diethylpyrocarbonate-treated water. The resultant total RNA was used for RNA-seq analyses conducted by Novogene (China). Read quality was filtered using Trimmomatic version 0.39 [13] and mapped to the reference genome of NGC7 (Genbank accession, BAAFZY010000001.1) by Bowtie2 version 2.5.0 [14] using Galaxy platform [15]. Relative transcript abundance was calculated as transcripts per million (TPM).

Preparation of purified VanR2 protein

A DNA fragment carrying *vanR2* was amplified by PCR using NGC7 genomic DNA as the template. The PCR product was cloned into the NdeI-BamHI sites of pET-21a(+) to construct pET-vanR2 with a His-tag fused to the 3′ end of *vanR2*. *E. coli* BL21(DE3) cells harboring pET-vanR2 were pre-cultured in LB and then inoculated into the fresh LB at a 100-fold dilution. The culture was incubated at 30°C until the OD_{600} reached 0.4–0.5, then 1 mM isopropyl-β-D-thiogalactopyranoside was added to induce the T7 promoter, and further incubated for 4 hours. The cells were harvested, washed twice with 50 mM phosphate buffer (pH 7.4), and resuspended in the same buffer. Cells were disrupted using a Q125 ultrasonic disintegrator (Qsonica, USA), followed by centrifugation (19,000 g, 15 min, 4°C) to separate the soluble and insoluble fractions. The supernatant was used as the soluble fraction, while the pellet was resuspended in 50 mM phosphate buffer (pH 7.4) as the insoluble fraction. The soluble fraction was applied to His SpinTrap™ TALON Co^{2+} affinity column (Cytiva, USA) pre-equilibrated with 50 mM phosphate buffer (pH 7.4) containing 500 mM NaCl. After centrifugation (100 g, 1min, 4°C), the column was washed twice with 50 mM phosphate buffer (pH 7.4) containing 500 mM NaCl and 20 mM imidazole. His tag-fused VanR2 was then eluted with 50 mM phosphate buffer (pH 7.4) containing 500 mM NaCl and 500 mM imidazole. Protein concentration was determined by the Bradford method [16]. The gene expression and purity of VanR2 were analyzed by sodium dodecyl sulphate polyacrylamide (12%) gel electrophoresis (SDS-PAGE), and protein bands in gels were visualized by staining with Coomassie Brilliant Blue.

Table S1. Strains and plasmids used in this study

Strain or plasmid	Relevant characteristic(s)[a]	Reference or Source
Strains		
Pseudomonas sp.		
NGC7	Wild type; Ap^{r}, Km^{r}	[17]
NGC715 (Δ*aph*)	NGC7 derivative; ΔPSN_1511 (*aph*); Ap^{r}	[12]
NGC721 (Δ*vanA2B2 mgaAB vanA1B1*)	NGC715 derivative; Δ*aph* PSN_2696-PSN_2691 (*vanA2B2 mgaAB vana1B1*); Ap^{r}	This study
NHC731 (Δ*galR*)	NGC715 derivative; Δ*aph* PSN_2703 (*galR*); Ap^{r}	This study
NGC733	NGC715 derivative; Δ*aph* PSN_2684; Ap^{r}	This study
NGC734	NGC715 derivative; Δ*aph* PSN_2685; Ap^{r}	This study
NGC735 (Δ*mgaD*)	NGC715 derivative; Δ*aph* PSN_2687 (*mgaD*); Ap^{r}	This study
NGC736	NGC715 derivative; Δ*aph* PSN_2690; Ap^{r}	This study
NGC737 (Δ*galC*)	NGC715 derivative; Δ*aph* PSN_2701 (*galC*); Ap^{r}	This study
NGC739	NGC715 derivative; Δ*aph* PSN_2683; Ap^{r}	This study
NGC740 (Δ*vanR2*)	NGC715 derivative; Δ*aph* PSN_2697 (*vanR2*); Ap^{r}	This study
NGC742	NGC715 derivative; Δ*aph* PSN_2686; Ap^{r}	This study
NGC743	NGC715 derivative; Δ*aph* PSN_2688; Ap^{r}	This study
NGC746 (Δ*fdhA2*)	NGC715 derivative; Δ*aph* PSN_2699 (*fdhA2*); Ap^{r}	This study
NGC749	NGC715 derivative; Δ*aph* PSN_2698; Ap^{r}	This study
NGC750 (Δ*galD*)	NGC715 derivative; Δ*aph* PSN_2700 (*galD*); Ap^{r}	This study
NGC751 (Δ*mgaC*)	NGC715 derivative; Δ*aph* PSN_2689 (*mgaC*); Ap^{r}	This study
NGC752 (Δ*vanA2B2 mgaAB vanA1B1 vanA4B4*)	NGC721 derivative; Δ*aph vanA2B2 mgaAB vanA1B1* PSN_3754 (*vanA4*)-PSN_3755 (*vanB4*); Ap^{r}	This study
NGC753 (Δ*vanA2B2 mgaAB vanA1B1 vanA4B4 vanA3B3*)	NGC752 derivative; Δ*aph vanA2B2 mgaAB vanA1B1 vanA4B4* PSN_3259 (*vanA3*)-PSN_3260 (*vanB3*); Ap^{r}	This study
NGC754 (Δ*vanR*)	NGC715 derivative; Δ*aph* PSN_3756 (*vanR*); Ap^{r}	This study
NGC755 (Δ*mgaAB*)	NGC715 derivative; Δ*aph* PSN_2693 (*mgaA*)-PSN_2694 (*mgaB*); Ap^{r}	This study
NGC756 (Δ*vanA1B1*)	NGC715 derivative; Δ*aph* PSN_2692 (*vanA1*)-PSN_2691 (*vanB1*); Ap^{r}	This study
NGC787 (Δ*galB*)	NGC715 derivative; Δ*aph* PSN_2702 (*galB*); Ap^{r}	This study
NGC7151 (Δ*frmAC*)	NGC715 derivative; Δ*aph* PSN_1646 (*frmA*)-PSN_1647 (*frmC*); Ap^{r}	This study
NGC7155 (Δ*fdhA2 frmAC*)	NGC746 derivative; Δ*aph fdhA2 frmAC*; Ap^{r}	This study
NGC7223 (Δ*vanA2B2*)	NGC715 derivative; Δ*aph* PSN_2695 (*vanA2*)-PSN_2696 (*vanB2*); Ap^{r}	This study
E. coli		
BL21(DE3)	F^{-} *ompT hsdS*$_{B}$(r$_{B}^{-}$ m$_{B}^{-}$) *gal dcm* (DE3); T7 RNA polymerase gene under the control of the *lacUV5* promoter	[18]
NEB 10β	Δ(*ara-leu*) *7697 araD139 fhuA* Δ*lacX74 galK16 galE15 e14- ϕ80*Δ*lacZ*Δ*M15 recA1 relA1 endA1 nupG rpsL* (Sm^{r}) *rph spoT1* Δ(*mrr-hsdRMS-mcrBC*)	New England Biolabs
Plasmids		
pET-21a(+)	expression vector; T7 promoter, Ap^{r}	Novagen
pK18*mobsacB*	suicide plasmid for gene disruption, Km^{r}	[19]
pSEVA2321	pBBR1 broad-host-range expression vector; P$_{lacIq}$; Km^{r}	[20]
pSEVA221	RK2 cloning vector, Km^{r}	[20]
pSEVAmNeon[b]	promoter probe vector; pSEVA221 with a synthesized fragment of *mNeongreen* gene	This study
pET-vanR2	pET21a with a PCR amplified fragment of *vanR2*	This study
pK18Δ*frmAC*	pK18*mobsacB* with a deletion cassette carrying up-and downstream regions of *frmAC*	This study
pK18Δ2683	pK18*mobsacB* with a deletion cassette carrying up-and downstream regions of PSN_2683	This study
pK18Δ2684	pK18*mobsacB* with a deletion cassette carrying up-and downstream regions of PSN_2684	This study
pK18Δ2685	pK18*mobsacB* with a deletion cassette carrying up-and downstream regions of PSN_2685	This study
pK18Δ2686	pK18*mobsacB* with a deletion cassette carrying up-and downstream regions of PSN_2686	This study
pK18Δ2686	pK18*mobsacB* with a deletion cassette carrying up-and downstream regions of PSN_2686	This study
pK18Δ2688	pK18*mobsacB* with a deletion cassette carrying up-and downstream regions of PSN_2688	This study
pK18Δ2689	pK18*mobsacB* with a deletion cassette carrying up-and downstream regions of PSN_2689	This study
pK18Δ2690	pK18*mobsacB* with a deletion cassette carrying up-and downstream regions of PSN_2690	This study
pK18Δ*vanA1B1*	pK18*mobsacB* with a deletion cassette carrying up-and downstream regions of *vanA1B1*	[12]
pK18Δ*vanA2B2* 2694-2693 *vana1B1*	pK18*mobsacB* with a deletion cassette carrying up-and downstream regions of *vanB2* and *vanB1*, respectively	This study
pK18Δ2694-2693	pK18*mobsacB* with a deletion cassette carrying up-and downstream regions of 2694-2693	This study

pK18Δ*vanA2B2*_2	pK18*mobsacB* with a deletion cassette carrying up-and downstream regions of *vanA2B2*	[12]
pK18Δ*vanR2*	pK18*mobsacB* with a deletion cassette carrying up-and downstream regions of *vanR2*	This study
pK18Δ2698	pK18*mobsacB* with a deletion cassette carrying up-and downstream regions of PSN_2698	This study
pK18Δ2699	pK18*mobsacB* with a deletion cassette carrying up-and downstream regions of PSN_2699	This study
pK18Δ2702	pK18*mobsacB* with a deletion cassette carrying up-and downstream regions of PSN_2702	This study
pK18Δ2701	pK18*mobsacB* with a deletion cassette carrying up-and downstream regions of PSN_2701	This study
pK18Δ2700	pK18*mobsacB* with a deletion cassette carrying up-and downstream regions of PSN_2700	This study
pK18Δ*galR*	pK18*mobsacB* with a deletion cassette carrying up-and downstream regions of *galR*	This study
pK18Δ*vanR*	pK18*mobsacB* with a deletion cassette carrying up-and downstream regions of *vanR*	This study
pK18Δ*vanA3B3*	pK18*mobsacB* with a deletion cassette carrying up-and downstream regions of PSN_3259-3260	[12]
pK18Δ*vanA4B4*	pK18*mobsacB* with a deletion cassette carrying up-and downstream regions of PSN_3754-3755	[12]
pvanA1B1	pSEVA2321 with PCR amplified fragment of *vanA1B1* and upstream region of *vanA1B1*	This study
pvanA2B2	pSEVA2321 with PCR amplified fragment of *vanA2B2* and upstream region of *vanA2B2*	This study
p2694-2693	pSEVA2321 with PCR amplified fragment of PSN_2694-2693 and upstream region of PSN_2694-2693	This study
p2689	pSEVA2321 with PCR amplified fragment of PSN_2689 and upstream region of PSN_2689	This study
p2687	pSEVA2321 with PCR amplified fragment of PSN_2687 and upstream region of PSN_2687	This study
p2688	pSEVA2321 with PCR amplified fragment of PSN_2688 and upstream region of PSN_2688	This study
p2702	pSEVA2321 with PCR amplified fragment of PSN_2702 and upstream region of PSN_2702	This study
p2701	pSEVA2321 with PCR amplified fragment of PSN_2701 and upstream region of PSN_2701	This study
p2700	pSEVA2321 with PCR amplified fragment of PSN_2700 and upstream region of PSN_2700	This study
pB2-1	pSEVAmNeon with a PCR amplified fragment of -220 to +1 relative to the *vanB2* start codon	This study
pB2-2	pSEVAmNeon with a PCR amplified fragment of -123 to +1 relative to the *vanB2* start codon	This study
pB2-3	pSEVAmNeon with a PCR amplified fragment of -92 to +1 relative to the *vanB2* start codon	This study
pB2-1_V1m	pB2-1 with the mutation at -156 to -153 (GGGA to AAAG)	This study
pB2-1_V2m	pB2-1 with the mutation at -92 to -89 (GGGA to AAAG)	This study
pB2-1_V3m	pB2-1with the mutation at -39 to -36 (GGG to AAAG)	This study
pB2-1_VAllm	pB2-1 with the mutation at -156 to -153 (GGGA to AAAG), -92 to -89 (GGGA to AAAG) and -39 to -36 (GGGA to AAAG)	This study<br>This study
pB2-2_-35m	pB2-2 with the mutation at -120 to -115 (TTGACT to CCAGTC)	This study
pB2-2_-10m	pB2-2 with the mutation at -97 to -92 (TAACAT to CGGTGC)	This study
pR2	pSEVAmNeon with a PCR amplified fragment of -220 to +1 relative to the *vanR2* start codon	This study
pGB	pSEVAmNeon with a PCR amplified fragment of -126 to +1 relative to the *galB* start codon	This study

[a]Km^r, Ap^r, and Sm^r resistance to kanamycin, ampicillin, and streptomycin respectively.

Table S2. Primers used in this study

Purpose	Primer	Nucleotide sequence (5′ to 3′)
Construction of plasmids		
pET-vanR2	pETR2_F	CTTTAAGAAGGAGATATACAATGAATCAACCCGCAGAAAT
	pETR2_R	ACGGAGCTCGAATTCGGATCCAGGGCCATACTGTGCCGCT
pK18Δ*frmAC*	frmAC_top_F	ATTCGAGCTCGGTACCCGGGCAAGTCCGAACTGCCAATCA
	frmAC_top_R	CAACGGTCTCCAGACAAGGG
	frmAC_bot_F	CCCTTGTCTGGAGACCGTTGTTCATCGAAGACCACCTGCG
	frmAC_bot_R	CAGGTCGACTCTAGAGGATCGCAGTTCCAGTTCGGTCATG
pK18Δ2683	2683_top_F	AGGTCGACTCTAGAGTCCACTGAGTACGCTTCTTC
	2683_top_R	GAGGTTCCCATTAGTCGCAT
	2683_bot_F	ATGCGACTAATGGGAACCTCGGTGCAAGTACGGGAACTAA
	2683_bot_R	CGGTACCCGGGGATCCGTCAGCCCCGTTTTACCTG
pK18Δ2684	2684_top_F	AGGTCGACTCTAGAGAGGCAACAGGGTTCGGTTCT
	2684_top_R	AAGGTTTCCGCCGATAAGCA
	2684_bot_F	TGCTTATCGGCGGAAACCTTGCAAAACAATCTGGCCTGGG
	2684_bot_R	CGGTACCCGGGGATCGATACGAGACACCGAAGCGA
pK18Δ2685	2685_top_F	AGGTCGACTCTAGAGGGCTCACCTGCTGATTCCAA
	2685_top_R	AGCAGTAATTGGGCGAAAGT
	2685_bot_F	ACTTTCGCCCAATTACTGCTCGCCCCCTGTAAGTAATCGC
	2685_bot_R	CGGTACCCGGGGATCGCGAGAGCTGCGCAAACAGG
pK18Δ2686	2686_top_F	AGGTCGACTCTAGAGTGCTCAAGTCGGCTACTCAG
	2686_top_R	GCTCCAAACGCTCTTCGATT
	2686_bot_F	AATCGAAGAGCGTTTGGAGCAGGTGACATTCTCCAGCTCG
	2686_bot_R	CGGTACCCGGGGATCCCCTTTTGCTGCTCGATCTA
pK18Δ2687	2687_top_F	AGGTCGACTCTAGAGCAACCGTGTAAAGGCAGAGC
	2687_top_R	CGCCAGTAGTGCTTCAATTC
	2687_bot_F	GAATTGAAGCACTACTGGCGCTCAGGGGTTGTGTAGGAAC
	2687_bot_R	CGGTACCCGGGGATCAACTGGTGCTCTGTTTTGCG
pK18Δ2688	2688_top_F	AGGTCGACTCTAGAGGGTCACGTCCAAGCTACATA
	2688_top_R	CGAGGTATTAGGTGTTGGCT
	2688_bot_F	AGCCAACACCTAATACCTCGGTTGAGGTCACGGGTGGATA
	2688_bot_R	CGGTACCCGGGGATCCCCGCATTTCATCGTTAGCA
pK18Δ2689	2689_top_F	AGGTCGACTCTAGAGATCTGATAAACGGCGACCAC
	2689_top_R	GTCGCACAGTAGTCCAGACA
	2689_bot_F	TGTCTGGACTACTGTGCGACTCAACCGTGTAAAGGCAGAG
	2689_bot_R	CGGTACCCGGGGATCTATCCACCCGTGACCTCAAC
pK18Δ2690	2690_top_F	AGGTCGACTCTAGAGGATAGCGGTGGCGTCGTAAT
	2690_top_R	GGCGACAATTCCCATCTGGA
	2690_bot_F	TCCAGATGGGAATTGTCGCCGCTGGCACTGAAACCGTGAA
	2690_bot_R	CGGTACCCGGGGATCTAGTTTTCCCTGCTGGTGCT
pK18Δ2694-2693	2694-2693_top_F	TAAAACGACGGCCAGTGCCAAAAAAGACGAAGGCAAGACC
	2694-2693_top_R	CTCACTGCTCTCCGGCTTTC
	2694-2693_bot_F	GAAAGCCGGAGAGCAGTGAGTAAAACTCACCAACATGCAG
	2694-2693_bot_R	ATTCGAGCTCGGTACCCGGGGTCTACCCAAGTCGTCAAAT
pK18Δ*vanA2B2* 2694-2693	2696-2691_top_F	TAAAACGACGGCCAGTGCCAAAAAATAGTGAACTCTGCGG
vanA1B1	2696-2691_top_R	GGGACAGCTCCTGAGTATTT
	2696-2691_bot_F	AAATACTCAGGAGCTGTCCCCGGTAATTGGCACCACACAT
	2696-2691_bot_F	ATTCGAGCTCGGTACCCGGGTAAAGGGTGTTGATTGACGT
pK18Δ2698	2698_top_F	AGGTCGACTCTAGAGACTACCCGAGCGTGACAAGG
	2698_top_R	TGTTGAAAGCACCTGTTCCA
	2698_bot_F	TGGAACAGGTGCTTTCAACAGCAACACAGAGGGCAGTTTC
	2698_bot_R	CGGTACCCGGGGATCAGGCACCCGCTGATGAATCA
pK18Δ*vanR2*	vanR2_top_F	AGGTCGACTCTAGAGAATTGACGTATCCCGACCCA
	vanR2_top_R	CCTTTGTTCGGTGATAGCCA
	vanR2_bot_F	TGGCTATCACCGAACAAAGGTGGTAACTCTACGCGAGCTG
	vanR2_bot_R	AGCTCGGTACCCGGGGCACTTCAGAATGCGTCTGT
pK18Δ*fdhA2*	fdhA2_top_F	AGGTCGACTCTAGAGAGCAAAGTGGCGATTATTGC
	fdhA2_top_R	TTCGATCTTATTGCCCCGAG

	fdhA2_bot_F	CTCGGGGCAATAAGATCGAACGCTACATCCTGATGGCAAG
	fdhA2_bot_R	CGGTACCCGGGGATCACGAACGATTGTGCGGATGG
pK18Δ2700	2700_top_F	AGGTCGACTCTAGAGCGAACCACATCAACCCGAGC
	2700_top_R	GTCAGGCTCCTACGTCATCG
	2700_bot_F	CGATGACGTAGGAGCCTGACGCTGCCGTGTGGAAATAGCC
	2700_bot_R	CGGTACCCGGGGATCATCTTCAGTCACGTACAGCC
pK18Δ2701	2701_top_F	ATTCGAGCTCGGTACCCGGGGCTGCTGCTCTGGTGATACTGACAGA
	2701_top_R	TTCTCCGTCACACG
	2701_bot_F	CGTGTGACGGAGAATCTGTCCTGCTCGAAAAGGGCTTGAA
	2701_bot_R	CAGGTCGACTCTAGAGGATCCGCACCATTCTTGAAGTCAGATTCGA
pK18Δ2702	2702_top_F	GCTCGGTACCCGGGGAATAAGGTAACGCCGCTCC
	2702_top_R	ATACCGATGTTACGCTTGGCGACCTGAACCTCCTTTGTATTGT
	2702_bot_F	ATACAAAGGAGGTTCAGGTCGCCAAGCGTAACATCGGTAT
	2702_bot_R	CAGGTCGACTCTAGAGGATCCTGCACAGGACACGGGAATA
pK18Δ*galR*	galR_top_F	AGGTCGACTCTAGAGCAGATCCCGCAATGGTCTTG
	galR_top_R	CAAAAGCTCCAGCCGATCAA
	galR_bot_F	TTGATCGGCTGGAGCTTTTGGGAAGCGGTTGATGGTCTTT
	galR_bot_R	AGCTCGGTACCCGGGCCCAGAGTAGTGACCGTAGA
pK18Δ*vanR*	vanR_top_F	ATTCGAGCTCGGTACCCGGGCTGGTGATCTGGTTGCTGTT
	vanR_top_R	TGCTGGATATCTGAAGCGCTCCAGGCAGTTTCCAGAGACA
	vanR_bot_F	TGTCTCTGGAAACTGCCTGGAGCGCTTCAGATATCCAGCA
	vanR_bot_R	CAGGTCGACTCTAGAGGATCTGACGAGGAACGGACATGAT
pvanA1B1	A1B1_F	CCCTAGGCCGCGGCCGCGCGGAAATGCGCTGACAAGAACG
	A1B1_F	CAGGTCGACTCTAGAGGATCTTAACGCAAAACTGGGTCGC
pvanA2B2	A2B2_F	CCCTAGGCCGCGGCCGCGCGAACAAGAACAAAATACTCAG
	A2B2_R	CAGGTCGACTCTAGAGGATCTCACTGCTCTCCGGCTTTCT
p2694-2693	2694-2693_F	CCCTAGGCCGCGGCCGCGCGCCCATAACAAGAAAGCCGGA
	2694-2693_R	CAGGTCGACTCTAGAGGATCTCAGCGCATTTCTGGAACAC
p2689	2689_F	ATTCGAGCTCGGTACCCGGGACCGTGAAATAAAATAGTGC
	2689_R	CAGGTCGACTCTAGAGGATCTTAGCTGCTATACGTATAGT
p2687	2687_F	ATTCGAGCTCGGTACCCGGGCAAGTTTTCTAATCATTCAG
	2687_R	CAGGTCGACTCTAGAGGATCCTAGCCAGGCATCTGCAACT
p2688	2688_F	ATTCGAGCTCGGTACCCGGGTCAAATAAAAACAATAATCG
	2688_R	CAGGTCGACTCTAGAGGATCTTAGAAAACTTGAGTGAGCC
p2702	2702_F	CCCTAGGCCGCGGCCGCGCGATCAACAACAATACAAAGGA
	2702_R	CAGGTCGACTCTAGAGGATCTCATGACAGATTCTCCGTCA
p2701	2701_F	CCCTAGGCCGCGGCCGCGCGCATCTTCCCGCGTGTGACGG
	2701_R	CAGGTCGACTCTAGAGGATCTCAGGCTCCTACGTCATCGG
p2700	2700_F	CCCTAGGCCGCGGCCGCGCGACAAATCAGCCGATGACGTA
	2700_R	CAGGTCGACTCTAGAGGATCCTATTTCCACACGGCAGCAG
pSEVAmNeon	mNeon_F	GCTCGGTACCCGGGGATCCTGAATTCAAAGGAGGTATATC
	mNeon_R	TGCCTGCAGGTCGACTCTAGGAATTCTCACTTGTACAGCT
pB2-1	pB2-1_F	CGCGAATTCGAGCTCGGTACCAGCGGGTGCCTTTGTTCGG
	pB2-1_R	TTTGAATTCAGGATCCCCGGCGGGACAGCTCCTGAGTATT
pB2-2	pB2-2_F	CGCGAATTCGAGCTCGGTACCGATTGACTTCGGATAGGGC
	pB2-1_R	TTTGAATTCAGGATCCCCGGCGGGACAGCTCCTGAGTATT
pB2-3	pB2-3_F	CGCGAATTCGAGCTCGGTACTGGGATCCCAATAGCGGCAAAA
	pB2-1_R	TTTGAATTCAGGATCCCCGGCGGGACAGCTCCTGAGTATT
pB2-2_-35m	pB2-2_-35m_F	CGCGAATTCGAGCTCGGTACCGACCAGTCTCGGATAGGGC
	pB2-1_R	TTTGAATTCAGGATCCCCGGCGGGACAGCTCCTGAGTATT
pB2-2_-10m	pB2-2_-10m_F	CGCGAATTCGAGCTCGGTACCGATTGACTTCGGATAGGGCCAACCA CGGTGCGGGATCCC
	pB2-1_R	TTTGAATTCAGGATCCCCGGCGGGACAGCTCCTGAGTATT
pR2	pR2_F	CGCGAATTCGAGCTCGGTACCGGGACAGCTCCTGAGTATT
	pR2_R	TTTGAATTCAGGATCCCCGGCAGCGGGTGCCTTTGTTCGG
pGB	pGB_F	CGCGAATTCGAGCTCGGTACCAAAAGCTCCAGCCGATCAA
	pGB_R	TTTGAATTCAGGATCCCCGGTGACCTGAACCTCCTTTGTA
Colony PCR (mutant confirmation)		
NGC721	2696-2691_conf_F	TGTCGGCTCTCTGGAAGATA

	2696-2691_conf_R	CGCACAGTAGTCCAGACAAA
NGC731	galR_conf_F	CCTACGTCATCGGCTGATTT
	galR_conf_R	TGCTAAAGCCTACATCGTGG
NGC733	2684_conf_F	GATCGTGCGTGAGGGTGAAA
	2684_conf_R	TGGGATGGTAGGAGCTTCTT
NGC734	2685_conf_F	TCGGACAGTTCAAAGATCGC
	2685_conf_R	CAGATTGTTTTGCCCCACCC
NGC735	2687_conf_F	GCATTCCCCTGATGGCTGAA
	2687_conf_R	GCATCGCACTCAGCAGTAAT
NGC736	2690_conf_F	GCACATTCCAACTCCGTCTA
	2690_conf_R	GACGTAGGTTGGATCTTGCT
NGC737	2701_conf_F	AGTCGATGTCTGCTCTGATG
	2701_conf_R	GACCACAGGCGACATTGAAA
NGC739	2683_conf_F	CTTATCGGCGGAAACCTTGT
	2683_conf_R	GAGGGCGTAGTAGCTGGACA
NGC740	2697_conf_F	TAGCCGCACACCAACACATC
	2697_conf_R	GACAGGATGCTAAACCCGGA
NGC742	2686_conf_F	AACTCCTTGGCTTTCACTGT
	2686_conf_R	TCATCAGTGGTTCTTCGGGA
NGC743	2688_conf_F	GCTCACGGCTTATTTCCTGC
	2688_conf_R	CTGGTGCTCTGTTTTGCGAA
NGC749	2698_conf_F	CTCGGGGCAATAAGATCGAA
	2698_conf_R	GAATGAACGCAGATCCCCGC
NGC746	2699_conf_F	AGTGTGGTCAAAGTCGGTCA
	2699_conf_R	CACCTCGTCAGCAACAACCG
NGC750	2700_conf_F	GAGGTTCAGGTCATGTCGAT
	2700_conf_R	CGGGTTTAGCATCCTGTCTT
NGC751	2689_conf_F	GCCAAAGTTCTGAGGAGAGT
	2689_conf_R	GCAACTGTAAAGATCCCCGT
NGC752	vanA4B4_conf_F	TGTACTGCTGAAACACCAGG
	vanA4B4_conf_R	CTTTGACGTGCTGGTGATGG
NGC53	vanA3B3_conf_F	GCTTGCTGCTTTGGTCCTTC
	vanA3B3_conf_R	AGAGTCGAAGTCGGGCTTGA
NGC754	vanR_conf_F	CTTTGACGTGCTGGTGATGG
	vanR_conf_R	CCTGACCCACGAAACCTACG
NGC755	2694-2693_conf_F	GCCTGAAGACACCGTTATCT
	2694-2693_conf_R	GACCTCAGCCATGCGTATGA
NGC756	vanA1B1_conf_F	GACCCTTCGTTCAAAAGCGT
	vanA1B1_conf_R	CGCACAGTAGTCCAGACAAA
NGC787	2702_conf_F	GTTCATGTCGAGTTCGTGGC
	2702_conf_R	CATTGATCTGCCTGCTTGGA
NGC7151	frmAC_conf_F	TATACGCCGCAAATGTTCCG
	frmAC_conf_R	GGCGAGAATCTGGTTGAACA
NGC7223	vanA2B2_conf_F	TGTCGGCTCTCTGGAAGATA
	vanA2B2_conf_R	GCGTCTACCCAAGTCGTCAA
RT-PCR analysis (target region)[a]		
1	2702-2701_F	ACAACTACGACCACCCGATG
1	2702-2701_R	CGATCAAGCCTTTAACGCCG
2	2701-27000_F	GTGTGGTCAAAGTCGGTCAA
2	2701-2700_R	CGCACCTTTTGGAGAAACGA
3	2700-2699_F	GGATAGCAACGGCACTCTCA
3	2700-2699_R	CCCACTTGAGCTGTAGTACG
4	2699-2698_F	GGAAGGCTACGGTGAGTTCG
4	2699-2698_R	TCTCGGGAAGACCAATTTCG
5	2696-2695_F	GATCACCTGGACTTCGTTGC
5	2696-2695_R	CATTGATGGCATGTTCACGC
6	2695-2694_F	GGGTATCTACGGTGTCCCAG
6	2695-2694_R	GACACCGATTGTTGGCACAT
7	2694-2693_F	CGTTGTCGTCGTTGGTACTG

7	2694-2693_R	GTCTTCCAACGATTCCCCGC
8	2693-2692_F	GGCACCTACGTCTTTGATCT
8	2693-2692_R	GTCCAGTAGCATTGAAGCGA
9	2692-2691_F	ACTCGATGTAGGTGTTGCCC
9	2692-2691_R	CGTACTACCGAATAGGAGCG
10	2691-2690_F	CTTGGCGTTGAGGTGGTAGT
10	2691-2690_R	CAATCCCGAACTGAGCAGCA
11	2690-2689_F	ACGGCTTATTTCCTGCTGAG
11	2690-2689_R	CAGAACGTGTTCAGCCATCA
12	2689-2688_F	TCGTTGCCCAGTCCTCATTG
12	2689-2688_R	CAAAGCCCGTAGCATCTCCA
13	2688-2687_F	TGACCCATTCGTTGCCGTAA
13	2688-2687_R	CCGCTTTGCCTTTGAGTTCC
14	2687-2686_F	CGGCTGAGTCGTCATTTTGT
14	2687-2686_R	TCAAGCGACTGACAAGAGCA
15	2686-2685_F	GTTCAGCTTGGGCGTTTCAA
15	2686-2685_R	GAATCATGCCTTCAGCCAAG
16	2685-2684_F	CGAACCAATCTTTGTCCCCC
16	2685-2684_R	ATTTCATCTTGGCGTGCCTC
17	2684-2683_F	AAGAGCGAACAACAGCGAAT
17	2684-2683_R	AAACGTCGCTTTGGTCTCAG
qRT-PCR analysis (target gene)		
16S rRNA	16S_qF	TAACACATGCAAGTCGAGCG
16S rRNA	16S_qR	TGCGGTATTAGCGTTCCTTT
PSN_2682	2682_qF	GCCAAGCAAGCCGAACTC
PSN_2682	2682_qR	GTTTGTTGCAGCCGTTCAAG
PSN_2683	2683_qF	CGCTAGAGAATCACGGCAATT
PSN_2683	2683_qR	TGCGCCGAGGCCTAATC
PSN_2684	2684_qF	GTCCCGAAGAACCACTGATGA
PSN_2684	2684_qR	AGTGCATCCGCGAGTTTCA
PSN_2685	2685_qF	TCAAGCGTCCTATCGAAATCG
PSN_2685	2685_qR	TTCGCGCTGTGCTTTGTATT
PSN_2686	2686_qF	TGGCGTTTGATCCTGAGTTCT
PSN_2686	2686_qR	TTGAAACGCCCAAGCTGAA
mgaD	2687_qF	GGCGGAGCGCGAATG
mgaD	2687_qR	CACCAGCGTCGTTCAGCAT
PSN_2688	2688_qF	AAGCTCAATGGCCGCAAA
PSN_2688	2688_qR	GCAACGAATGGGTCAGCAA
mgaC	2689_qF	CCTTGGAGATCATGCGCATA
mgaC	2689_qR	GTCTAGCAACGCGAGCCTTT
PSN_2690	2690_qF	TGCTGGTTTCATCACTGACTCAA
PSN_2690	2690_qR	TCCTGTAATTACGCGCATCAAC
vanB1	B1_qF	CGACCAGATCACCGCTCCTA
vanB1	B1_qR	AATTACGACGCCACCGCTAT
vanA1	A1_qF	TTCCCGCTTAACGCTTGGTA
vanA1	A1_qR	AATGCCGGCCAATTTCAG
mgaA	2693_qF	TCGCACGCGTTGTAAAACTC
mgaA	2693_qR	TTCCCCGCGCATGATG
mgaB	2694_qF	CCTCGCATGTGCCAACAA
mgaB	2694_qR	TGCCGGATCCTGTTGCTTA
vanA2	A2_qF	CTTATCCACCGCCGCATT
vanA2	A2_qR	AGCCAGGGTGCGGTACATT
vanB2	B2_qR	TGGCACGCGGGATCAT
vanR2	R2_qF	GCTTACGGAGTGATGAACCA
vanR2	R2_qR	GGTTTTCCTGCTGCTGTAAG
fdhA2	FD2_qF	CCGGTAAGGTCGAAGTTCAAAA
fdhA2	FD2_qR	CTTATTGCCCCGAGGATCCT
galB	GB_qF	CGCGCCGACAAAGAAACT
galB	GB_qR	CGGCTGAATCCGACGATAGA

vanA4	A4_qF	TGGACCGCTGGCAGATCT
vanA4	A4_qR	CGCCAACTTCGATTAGCACAT
vanB4	B4_qF	GTTGATACGCAGAACGACGG
vanB4	B4_qR	TGTTCAAGCACCTGCACCAC
frmA	FA_qF	AGAAACCCCTGGAAATCGTC
frmA	FA_qR	CAAGGATCGACGGGAAGATAC
EMSA probes[b]		
B2-1, B2-1_V1m, B2-1_V2m, B2-1_V3m, and B2-1_VAllm	B2-1_F	CAGCGGGTGCCTTTGTTCGG
	B2-1_R	CGGGACAGCTCCTGAGTATT
IR-VNone	VNone_F	CAAGAACAAAATACTCAGGAGCTG
	VNone_R	CATCAAGCGAGGTCAGCAGG
IR-V1	B2-1_F	CAGCGGGTGCCTTTGTTCGG
	IR-V1_R	GCCCTATCCGAAGTCAATCG
IR-V2	IR-V2_F	CGATTGACTTCGGATAGGGC
	IR-V2_R	TCAGGGTATCCGTAGCGTAA
IR-V3	IR-V3_F	GGATACCCTGACTGGGGATCCCAACAAGAACAAAATAC
	VNone_R	CATCAAGCGAGGTCAGCAGG

[a]The target regions correspond to the RT-PCR analysis shown in Figure 2A.

[b]Synthesized fragments, IR-V1m, IR-V2m, IR-V3m, and IR-VAllm, listed in Table S3, were used as PCR templates to amplify the B2-1_V1m, B2-1_V2m, B2-1_V3m, and B2-1_VAllm probes, respectively.

Table S3. Synthesized DNA fragments used in this study

Purpose	Nucleotide sequence (5′ to 3′)[a]
Fluorescent protein used for the construction of promoter probe vector	
mNeonGreen gene	AAAGGAGGTATATCATATGGTGAGCAAGGGCGAAGAGGACAACATGGCCAGTTT<br>GCCAGCGACCCATGAACTGCACATCTTCGGCTCCATTAACGGCGTAGACTTCGAC<br>ATGGTCGGCCAAGGAACCGGCAATCCGAACGACGGCTACGAAGAGCTCAACCTG<br>AAGAGCACGAAGGGGGATCTGCAGTTCAGCCCCTGGATCCTGGTTCCGCACATTG<br>GCTATGGGTTCCACCAGTATCTGCCGTATCCAGACGGCATGAGCCCCTTCCAAGC<br>AGCCATGGTGGATGGCTCAGGGTATCAGGTGCATCGCACGATGCAGTTCGAGGAC<br>GGTGCGTCGCTTACCGTCAACTACCGCTACACCTACGAAGGCTCCCACATCAAAG<br>GTGAAGCGCAGGTGAAAGGCACTGGCTTTCCTGCCGATGGTCCGGTCATGACGAA<br>CAGCTTGACTGCTGCGGATTGGTGCCGTTCGAAGAAGACCTACCCCAACGACAAG<br>ACCATCATCTCGACCTTCAAGTGGAGCTACACGACCGGCAATGGCAAACGCTACC<br>GGTCTACAGCCCGTACCACCTACACCTTCGCCAAACCGATGGCAGCCAACTACCT<br>GAAGAACCAGCCGATGTACGTGTTCCGCAAAACCGAGCTCAAGCACTCGAAGAC<br>CGAACTGAACTTCAAGGAGTGGCAGAAGGCCTTTACCGACGTGATGGGTATGGAC<br>GAGCTGTACAAGTGA
Construction of reporter plasmids and preparation of EMSA probes[b]	
IR-V1m	ACTAGCTCCATTTCAAATACCGCGAATTCGAGCTCGGTACCAGCGGGTGCCTTTGT<br>TCGGTGATAGCCAGGACTTTTAGCCCCGTTTAGGAAGATTCTGCCCTACAAAGTC<br>CCAAATGCCAAAAAAGCATGATTACCCGATTGACTTCGGATAGGGCCAACCATAA<br>CATGGGATCCCAATAGCGGCAAAAACCTGAAATTTACGCTACGGATACCCTGACT<br>GGGGATCCCAACAAGAACAAAATACTCAGGAGCTGTCCCGCCGGGGATCCTGAA<br>TTCAAACTAGCTCCATTTCAAATAC
IR-V2m	ACTAGCTCCATTTCAAATACCGCGAATTCGAGCTCGGTACCAGCGGGTGCCTTTGT<br>TCGGTGATAGCCAGGACTTTTAGCCCCGTTTAGGAAGATTCTGCCCTACGGGATC<br>CCAAATGCCAAAAAAGCATGATTACCCGATTGACTTCGGATAGGGCCAACCATAA<br>CATAAAGTCCCAATAGCGGCAAAAACCTGAAATTTACGCTACGGATACCCTGACT<br>GGGGATCCCAACAAGAACAAAATACTCAGGAGCTGTCCCGCCGGGGATCCTGAA<br>TTCAAACTAGCTCCATTTCAAATAC
IR-V3m	ACTAGCTCCATTTCAAATACCGCGAATTCGAGCTCGGTACCAGCGGGTGCCTTTGT<br>TCGGTGATAGCCAGGACTTTTAGCCCCGTTTAGGAAGATTCTGCCCTACGGGATC<br>CCAAATGCCAAAAAAGCATGATTACCCGATTGACTTCGGATAGGGCCAACCATAA<br>CATGGGATCCCAATAGCGGCAAAAACCTGAAATTTACGCTACGGATACCCTGACT<br>GAAAGTCCCAACAAGAACAAAATACTCAGGAGCTGTCCCGCCGGGGATCCTGAA<br>TTCAAACTAGCTCCATTTCAAATAC
IR-VAllm	ACTAGCTCCATTTCAAATACCGCGAATTCGAGCTCGGTACCAGCGGGTGCCTTTGT<br>TCGGTGATAGCCAGGACTTTTAGCCCCGTTTAGGAAGATTCTGCCCTACAAAGTC<br>CCAAATGCCAAAAAAGCATGATTACCCGATTGACTTCGGATAGGGCCAACCATAA<br>CATAAAGTCCCAATAGCGGCAAAAACCTGAAATTTACGCTACGGATACCCTGACT<br>GAAAGTCCCAACAAGAACAAAATACTCAGGAGCTGTCCCGCCGGGGATCCTGAA<br>TTCAAACTAGCTCCATTTCAAATAC

[a]DNA sequence including a ribosome binding site is underlined.

[b]IR-V1m, IR-V2m, IR-V3m, and IR-VAllm were used as PCR templates for the construction or preparation of reporter plasmids and EMSA probes corresponding to pB2-1_V1m/IR-V1m, pB2-1_V2m/IR-V2m, pB2-1_V3m/IR-V3m, and pB2-1_VAllm/IR-VAllm, respectively.

Table S4. Genes in the vicinity of *vanA1B1* and their transcript levels based on RNA-seq analysis

Locus tag (gene)	Product	Most similar protein[a]	Accession No.	Identity[b] (%)	TPM[c] Glc	TPM[c] SA	Ratio of TPM SA/Glc
PSN_2703 (*galR*)	LysR family transcriptional regulator	GalR	Q88JX7.1	49	20	32	1.6
PSN_2702 (*galB*)	4-oxalmesaconate hydratase GalB	GalB	Q88JX8.1	82	10	2583	250
PSN_2701 (*galC*)	4-carboxy-4-hydroxy-2-oxoadipate aldolase/oxaloacetate decarboxylase GalC	GalC	Q88JX9.1	68	21	1990	94
PSN_2700 (*galD*)	4-oxalomesaconate tautomerase GalD	GalD	Q88JY0.1	62	8	1151	144
PSN_2699 (*fdhA2*)	glutathione-independent formaldehyde dehydrogenase	FdhA	P46154.1	94	22	4719	212
PSN_2698	PAS domain-containing methyl-accepting chemotaxis protein	BdlA	Q9I3S1.1	19	3	289	107
PSN_2697 (*vanR2*)	GntR family transcriptional regulator	YdhC	O05494.1	23	41	77	1.9
PSN_2696 (*vanB2*)	PDR/VanB family oxidoreductase	TsaB2	Q9AHG2.1	38	12	1182	100
PSN_2695 (*vanA2*)	aromatic ring-hydroxylating dioxygenase subunit alpha	TsaM1	P94679.1	31	17	4894	292
PSN_2694 (*mgaB*)	gallate dioxygenase	LigB	P22636.1	41	14	5356	378
PSN_2693 (*mgaA*)	gallate dioxygenase	GalA	Q88JX5.1	17	24	9016	377
PSN_2692 (*vanA1*)	VA *O*-demethylase oxygenase subunit	VanA	O05616.1	42	18	5500	313
PSN_2691 (*vanB1*)	PDR/VanB family oxidoreductase	CntB	D0C9N8.1	28	15	4306	284
PSN_2690	MFS transporter	GalT	E8ZB61.1	29	1	968	800
PSN_2689 (*mgaC*)	α/β fold hydrolase	RutD	B0SW62.1	22	5	3542	676
PSN_2688	transporter	N/A	-	-	2	2648	1215
PSN_2687 (*mgaD*)	nuclear transport factor 2 family protein	N/A	-	-	8	6655	820
PSN_2686	AraC family transcriptional regulator	FeaR	Q47129.1	18	83	555	6.7
PSN_2685	carotenoid oxygenase family protein	LSD-I	Q53353.1	33	6	586	91
PSN_2684	aldehyde dehydrogenase	PatD	Q6D6Y7.1	38	9	320	37
PSN_2683	OmpP1/FadL family transporter	N/A	-	-	6	136	23
PSN_2682	hypothetical protein	N/A	-	-	81	132	1.6

[a]Proteins were searched using the BLAST-P program with the UniProtKB database. If no significant similarity is found, it is indicated by not applicable (N/A).

[b]Amino acid sequence identity was calculated by the EMBOSS Needle pairwise global alignment program.

[c]RNA-seq analyses were performed using total RNA isolated from NGC7 cells grown in MMx-3 medium containing 5 mM Glc or 5 mM SA. Transcript levels are expressed as transcripts per million (TPM).

Table S5. Putative methanol, HCHO, and HCOOH dehydrogenases in NGC7 and their transcript levels based on RNA-seq analysis.

Locus tag (gene)	Product	Most similar protein from KT2440	Accession No.	Identity[a] (%)	TPM[b] Glc	TPM[b] SA	Ratio of TPM SA/Glc
methanol dehydrogenase							
PSN_2558 (*pedE*)	PQQ-dependent quinoprotein ethanol dehydrogenase	PedE	Q88JH5	97	4	244	70
PSN_2563 (*pedH*)	PQQ-dependent alcohol dehydrogenase PedH	PedH	Q88JH0	96	2	193	85
PSN_2569 (*yiaY*)	Fe containing alcohol dehydrogenase	YiaY	AAN67799.1	99	24	130	5.4
PSN_3827 (*adhP*)	alcohol dehydrogenase	AdhP	Q88G86	92	5	4	0.7
HCHO dehydrogenase							
PSN_0408 (*fdhA*)	glutathione-independent formaldehyde dehydrogenase	FdhA	Q88R06	98	15	47	3.1
PSN_1646 (*frmA*)	glutathione-dependent formaldehyde dehydrogenase FrmA	FrmA	AAN67237.1	97	106	211	2.0
PSN_1647 (*frmC*)	S-formylglutathione hydrolase FrmC	FrmC	AAN67238.1	97	49	128	2.6
PSN_2564 (*aldB-II*)	aldehyde dehydrogenase family protein	AldB-II	Q88JG7	95	4	251	57
PSN_2699 (*fdhA2*)	glutathione-independent formaldehyde dehydrogenase	FdhA	Q88R06	98	22	4719	212
PSN_5641 (*fdhB*)	zinc-type alcohol dehydrogenase-like protein YbdR	FdhB	AAN69564.1	92	17	194	11
HCOOH dehydrogenase							
PSN_0320	putative formate dehydrogenase oxidoreductase protein	PP_0256	AAN65887.1	95	38	90	2.4
PSN_0321 (*fdhD*)	sulfur carrier protein FdhD	FdhD	AAN65888.1	94	50	136	2.7
PSN_0593 (*fdoG*)	Formate dehydrogenase N alpha subunit	FdoG	AMM02774.1	98	280	462	1.6
PSN_0594 (*fdoH*)	Formate dehydrogenase N beta subunit	FdoH	AAN66119.1	98	46	98	2.1
PSN_0595 (*fdoI*)	Formate dehydrogenase O gamma subunit	FdoI	AAN66120.1	90	44	90	2.1
PSN_0596 (*fdhE*)	Formate dehydrogenase formation protein FdhE	FdhE	AAN66121.1	83	41	98	2.4
PSN_2148 (*fmdE*)	NAD-dependent formate dehydrogenase gamma subunit	FmdE	AAN67796.1	88	40	154	3.9
PSN_2149 (*fmdF*)	NAD-dependent formate dehydrogenase beta subunit	FmdF	AAN67797.1	93	17	62	3.7
PSN_2150 (*fmdG*)	NAD-dependent formate dehydrogenase alpha subunit	FmdG	AAN67798.1	96	20	63	3.1
PSN_2151 (*fmdH*)	NAD-dependent formate dehydrogenase delta subunit	FmdH	Q88JG9	90	134	87	0.7
PSN_4723	putative formate dehydrogenase oxidoreductase protein	PP_4596	AAN70169.1	90	6049	1597	0.3

[a]Amino acid sequence identity was calculated by the EMBOSS Needle pairwise global alignment program.

[b]RNA-seq analyses were performed using total RNA isolated from NGC7 cells grown in MMx-3 medium containing 5 mM Glc or 5 mM SA. Transcript levels are expressed as transcripts per million (TPM).

Table S6. Enzymes used to investigate the conservation of SA catabolism genes

Enzyme	Product	Organism	Accession No.
VanA1	SA *O*-demethylase oxygenase subunit	*Pseudomonas* sp. NGC7	GAB1616953.1
VanB1	SA *O*-demethylase oxidoreductase subunit	*Pseudomonas* sp. NGC7	GAB1616952.1
MgaA	putative 3MGA dioxygenase α subunit	*Pseudomonas* sp. NGC7	GAB1616954.1
MgaB	putative 3MGA dioxygenase β subunit	*Pseudomonas* sp. NGC7	GAB1616955.1
MgaC	putative CHMOD hydrolase	*Pseudomonas* sp. NGC7	GAB1616950.1
MgaD	putative CHMA tautomerase	*Pseudomonas* sp. NGC7	GAB1616948.1
GalD	OMA tautomerase	*Pseudomonas* sp. NGC7	GAB1616961.1
GalB	2-keto-4-carvoxy-3-hexenedionic acid hydratase	*Pseudomonas* sp. NGC7	GAB1616963.1
GalC	4-carboxy-4-hydroxy-2-oxoadipic acid aldolase	*Pseudomonas* sp. NGC7	GAB1616962.1
LigA	protocatechuic acid 4,5-dioxygenase α subunit	*S. lignivorans* SYK-6	BAK65926.1
LigB	protocatechuic acid 4,5-dioxygenase β subunit	*S. lignivorans* SYK-6	BAK65925.1
LigM	VA/3MGA *O*-demethylase	*S. lignivorans* SYK-6	BAK65949.1
DesZ	3MGA 3,4-dioxygenase	*S. lignivorans* SYK-6	BAK66578.1
DesB	gallic acid dioxygenase	*S. lignivorans* SYK-6	BAK65008.1
LigI	PDC hydrolase	*S. lignivorans* SYK-6	BAK65932.1
LigU	(4E)-oxalomesaconate delta-isomerase	*S. lignivorans* SYK-6	BAK65931.1
LigJ	2-keto-4-carboxy-3-hexenedioate hydratase	*S. lignivorans* SYK-6	BAK65927.1
LigK	4-carboxy-4-hydroxy-2-oxoadipate aldolase	*S. lignivorans* SYK-6	BAK65930.1
PmdD	PDC hydrolase	*Comamonas* sp. E6	GAO68735.1
PmdU	OMA tautomerase	*Comamonas* sp. E6	GAO68739.1
PmdE	4-oxalomesaconate hydratase	*Comamonas* sp. E6	GAO68737.1
PmdF	4-carboxy-4-hydroxy-2-oxoadipate aldolase	*Comamonas* sp. E6	GAO68736.1
DesC	CHMOD methylesterase	*N. aromaticivorans* DSM12444	WP_011446504.1
DesD	CHMOD methylesterase	*N. aromaticivorans* DSM12444	WP_011446505.1
VanA	VA *O*-demethylase oxygenase subunit	*P. putida* KT2440	Q88GI6
VanB	VA *O*-demethylase oxidoreductase	*P. putida* KT2440	Q88GI5
GalA	gallic acid dioxygenase	*P. putida* KT2440	Q88JX5.1

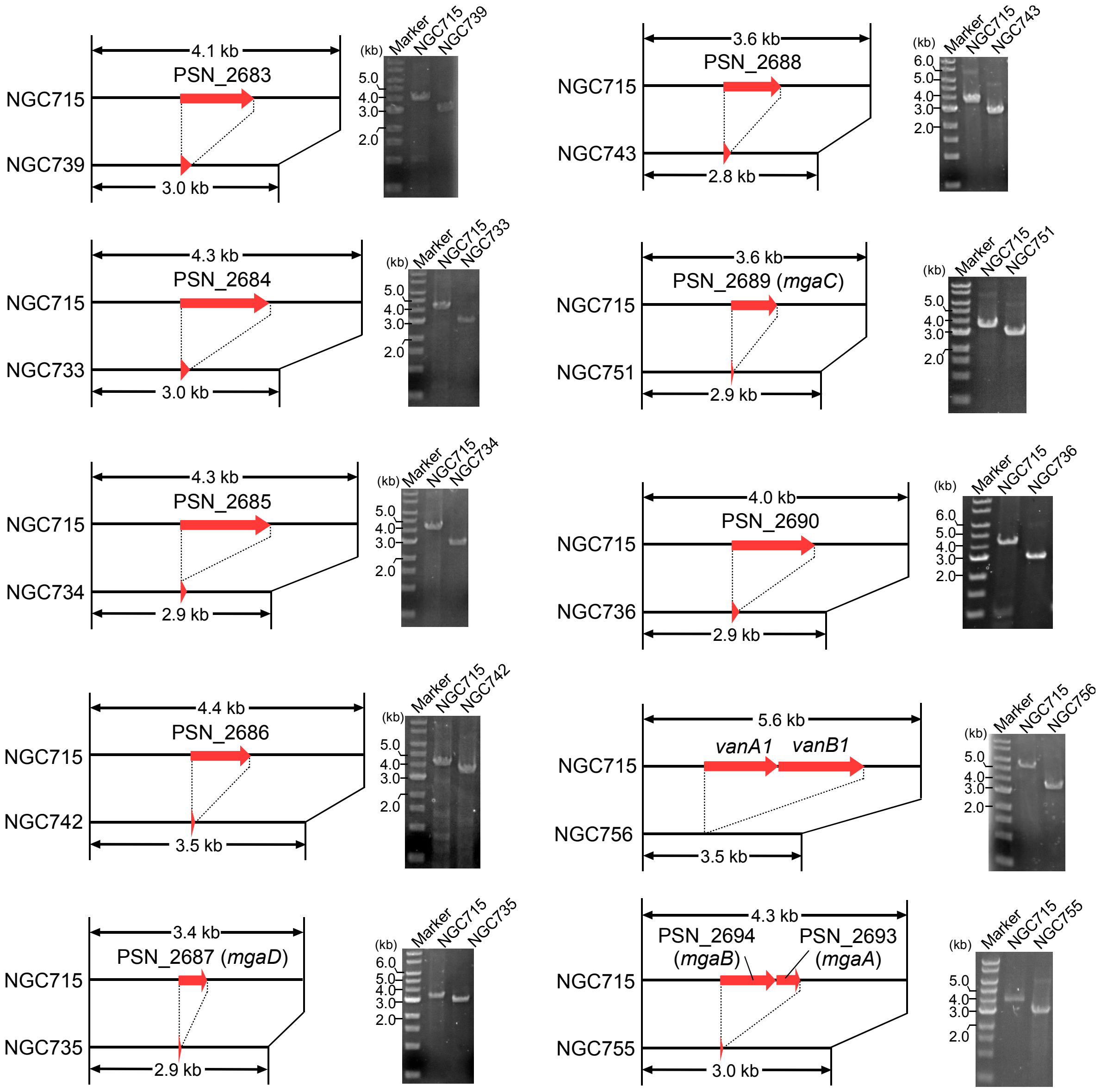

Figure S1. Construction of the gene deletion mutants.

The schematic representations of the gene deletion are shown on the left. The target gene retains 12-153 bp at both the 5′ and 3′ ends or complete deletion, which are joined together after deletion. When multiple genes are deleted simultaneously, 12-50 bp at the 5′ end of the most upstream gene and the 3′ end of the most downstream gene remain or complete deletion and are joined together. Colony PCR analyses for the confirmation of gene deletion are shown on the right. M, molecular size markers.

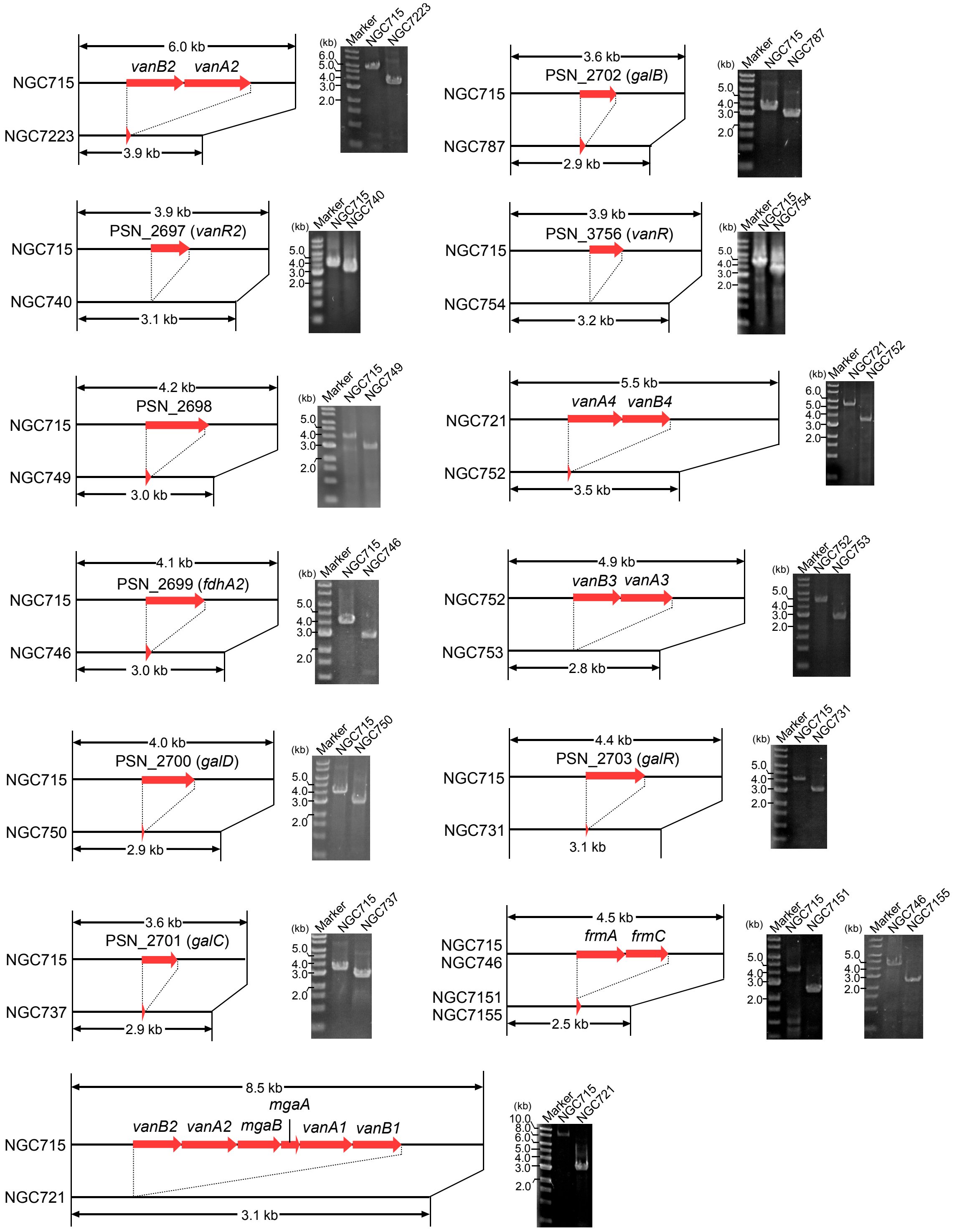

Figure S1-*Continued*.

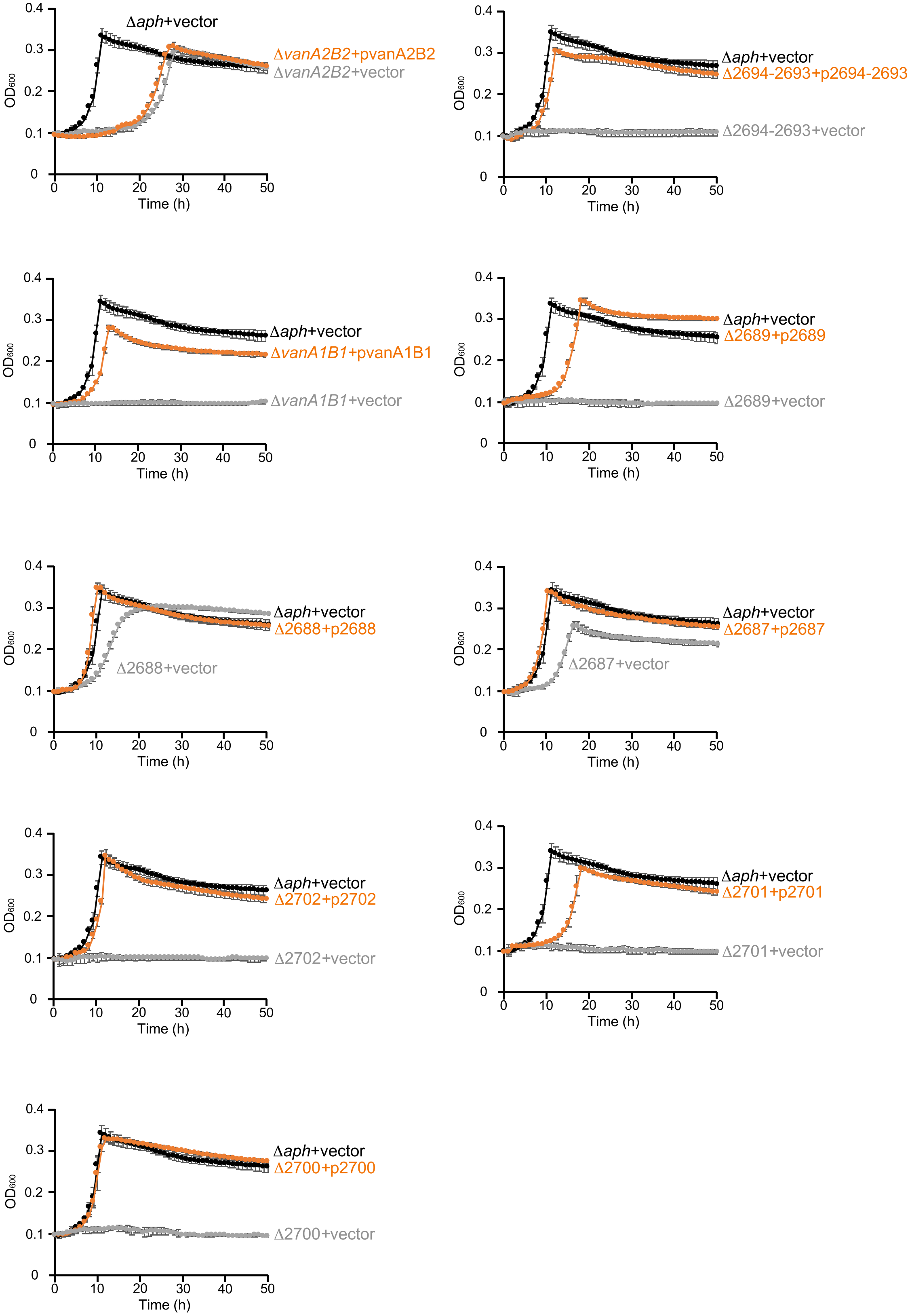
Δaph+vector
ΔvanA2B2+pvanA2B2
ΔvanA2B2+vector
Δaph+vector
Δ2694-2693+p2694-2693
Δ2694-2693+vector
Δaph+vector
ΔvanA1B1+pvanA1B1
ΔvanA1B1+vector
Δaph+vector
Δ2689+p2689
Δ2689+vector
Δaph+vector
Δ2688+p2688
Δ2688+vector
Δaph+vector
Δ2687+p2687
Δ2687+vector
Δaph+vector
Δ2702+p2702
Δ2702+vector
Δaph+vector
Δ2701+p2701
Δ2701+vector
Δaph+vector
Δ2700+p2700
Δ2700+vector
OD600
Time (h)
0
0.1
0.2
0.3
0.4
10
20
30
40
50

Figure S2. Growth complementation of SA catabolic gene mutants.

Cells of each strain were grown in MMx-3 medium containing 5 mM SA. Cell growth was monitored by measuring OD_{600}. Growth of Δ*aph*, a kanamycin resistance gene mutant of NGC7, harboring pSEVA2321 vector, mutants harboring pSEVA2321, and mutants harboring each complementary plasmid are indicated by black, gray, and orange, respectively. Complementary plasmids (Table S1) were constructed by introducing the mutated gene into the downstream of P_{lacIq} constitutive promoter. Each value represents the mean ± standard deviation (error bars) from three independent experiments.

(A)

```
PSN_2694       1 MAKIVGAIGTSHVPTIGVAYDKGKQQDPAWAPLFDGYKPVASWLKDKKVD     50

GalA           1 --------------------------------------------------      0

PSN_2694      51 VLVFFYNDHCTTFFFDMYPMFALGVGERFQVADEGAGLRPLPPIRGDVAL    100
                                             .:||||...|.|||:.|..||
GalA           1 -----------------------------AMADEGGNPRDLPPVGGHAAL     21

PSN_2694     101 QAHIAESLANDEFDITIFQNKPIDHGCAAPLPLLWPHEPDWPGVVVPIAI    150
                 ..||.:||..||||::.|::||:|||..:|:..|.|.:..||..:||:.:
GalA          22 SRHIGQSLMADEFDMSFFRDKPLDHGFFSPMSALLPCDESWPVQIVPLQV     71

PSN_2694     151 NVLQYPLPTAQRCYRLGQAIRKAVESYPEDLNVVVVGTGGLSHQIHGERT    200
                 .|||.|:|||:|||:||||:|:|:|||||||.|.:|.|||:|||:||||.
GalA          72 GVLQLPIPTARRCYKLGQALRRAIESYPEDLKVAIVATGGVSHQVHGERC    121

PSN_2694     201 GFNNTEWDMEFLELLQHAPEKLTQMTHADYVRLGGAESVEQIMWLAMRGA    250
                 ||||.|||.:||:||.:.|::||:||.|:|..|||.|..|.|.||.|||.
GalA         122 GFNNPEWDAQFLDLLVNDPQRLTEMTLAEYATLGGMEGAEVITWLIMRGT    171

PSN_2694     251 VEGPLNKIHQNYYLATTTAMTVVLYETVTETADAQAIA------------    288
                 :...:.:.||:|||.:.|.:..:|.|...:...|....
GalA         172 LSANVERKHQSYYLPSMTGIATLLLENRDQALPAPVNERHRQHMQHQLAG    221
```

(B)

```
PSN_2693       1 -------------MNPQVIGMQDLGGTYVFDLRVSNKALELNRFFWNMI     36
                              |..|:.|.:.|.|||.:.|..|.|...||:|...||
GalA         201 QALPAPVNERHRQHMQHQLAGAEQLEGTYPYTLERSAKGYRLNKFLHRMI    250

PSN_2693      37 TPQWRERFLEDEEQLMTDAELTEAEKVLVRARDWLGLVQYGANFFVVEKF     86
                 .||||:|||.:.|.|..:|.|:|.|..|:|.|||.||:.||..|||:||.
GalA         251 EPQWRQRFLSEPEALYREAGLSEEESDLLRRRDWRGLIHYGVIFFVLEKL    300

PSN_2693      87 ARVVKLTNMQVYAIMRGESLEDFLKTRRVPEMR---------    119
                 ..|:.::|:.:||.|||:|:|||:|||. .::|
GalA         301 GAVLGVSNLDIYAAMRGQSIEDFMKTRN-QQVRYSVAGKAPN    341
```

(C)

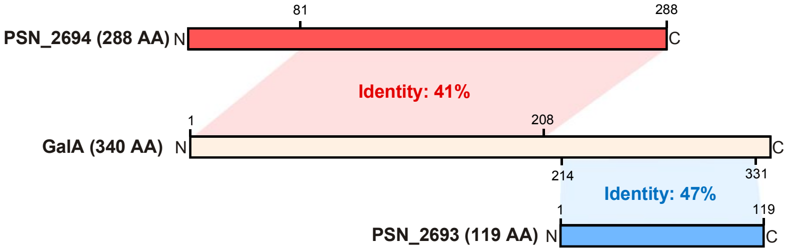

Figure S3. Amino acid sequence of PSN_2693 (MgaA) and PSN_2694 (MgaB) compared to GalA from *P. putida* KT2440.

Pairwise amino acids sequence alignment between PSN_2694 (MgaB) and gallic acid dioxygenase (GalA) (A) and between PSN_2693 (MgaA) and GalA (B). The accession numbers of the proteins used for the alignments are listed in Table S6. (C) Schematic diagram of the regions where PSN_2694 and PSN_2693 exhibiting homology to GalA. Amino acid sequence identities were analyzed using residues 81-288 of PSN_2694 and 1-208 of GalA and residues 1-119 of PSN_2693 and 214-331 of GalA. Amino acid sequence identity was calculated by the EMBOSS Needle pairwise global alignment program.

(A)

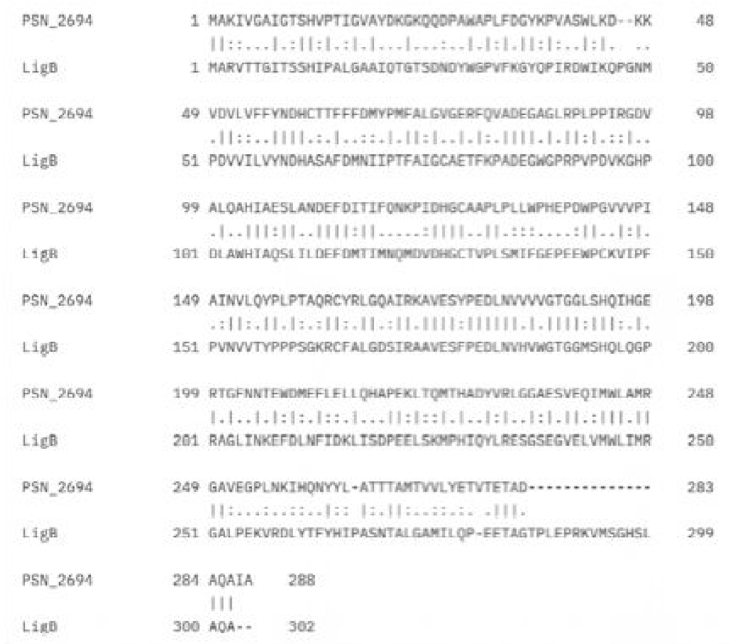

(B)

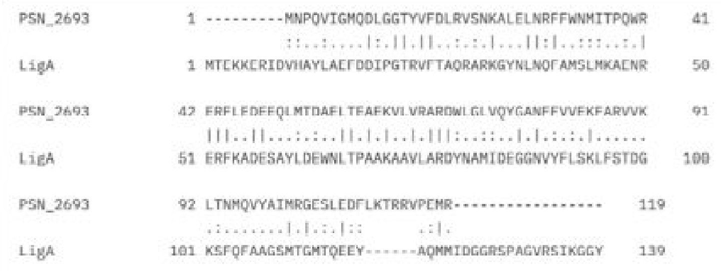

Figure S4. Amino acid sequence of PSN_2693 (MgaA) and PSN_2694 (MgaB) compared to LigA and LigB, respectively, from *S. lignivorans* SYK-6.

Pairwise amino acids sequence alignment between PSN_2694 (MgaB) and protocatechuic acid 4,5-dioxygenase β subunit (LigB) (A) and between PSN_2693 (MgaA) and protocatechuic acid 4,5-dioxygenase α subunit (LigA) (B). The accession numbers of the proteins used for the alignments are listed in Table S6.

(A) PSN_2689 from NGC7

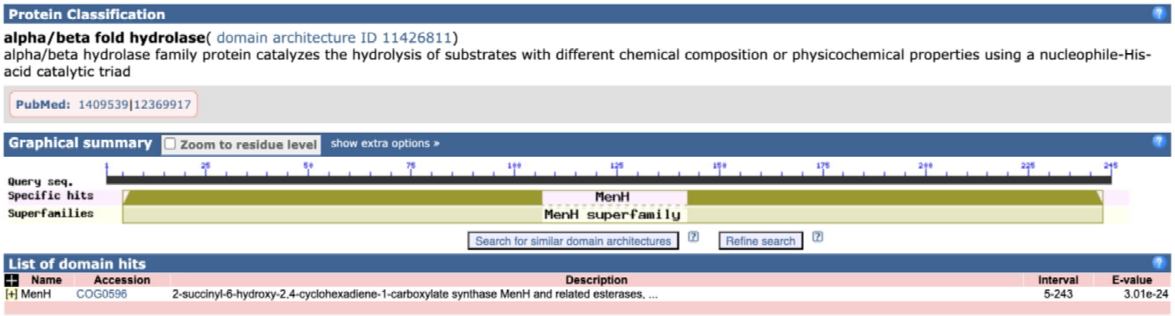

(B) DesC from *N. aromaticivorans* DSM 12444

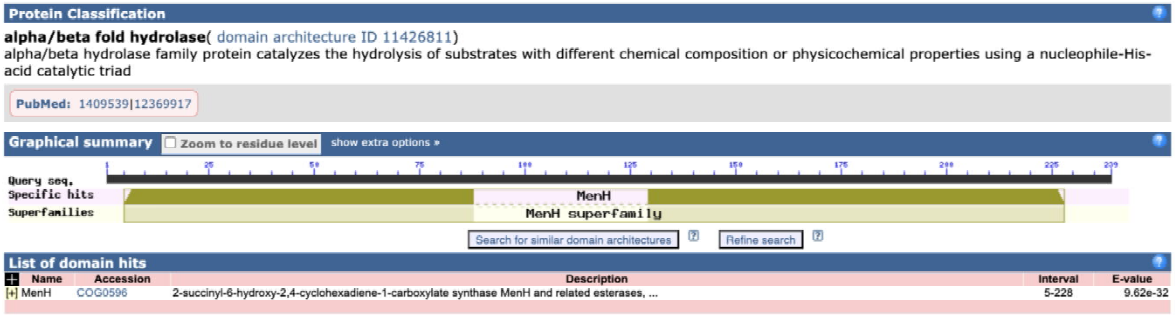

Figure S5. Comparison of the predicted domains of PSN_2689 (MgaC) and DesC from *N. aromaticivorans* DSM 12444.

The predicted domain architectures of PSN_2689 (A) and DesC (B) are shown. A conserved domain search indicated that both PSN_2689 and DesC are classified in the α/β fold hydrolase family (domain architecture ID: 11426811) with MenH domain (COG0596).

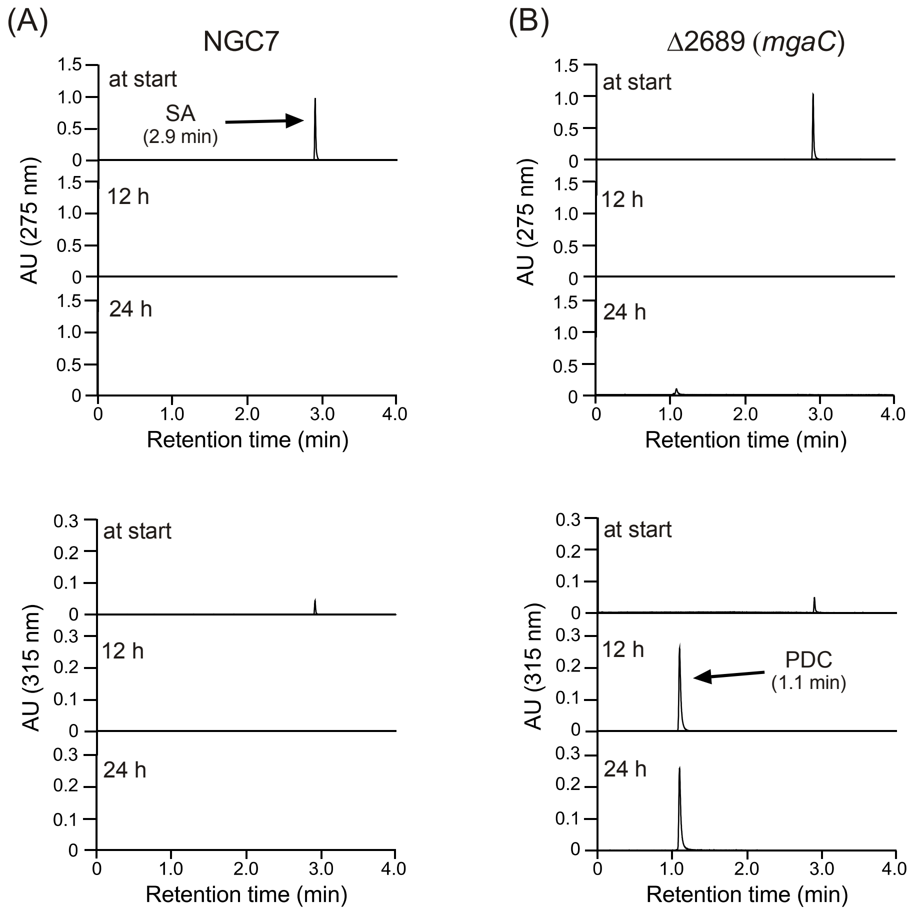

Figure S6. HPLC analysis of SA degradation products by NGC7 and Δ2689 (*mgaC*).

HPLC chromatograms of culture supernatants from NGC7 (A) and Δ2689 (*mgaC*). Chromatograms at 275 nm (top) and 315 nm (bottom) for the detection of SA and PDC are shown. Cells of NGC7 and Δ2689 (*mgaC*) were grown in MMx-3 medium containing 5 mM Glc and 5 mM SA. Samples were collected at the start, after 12 h, and after 24 h and then analyzed by HPLC.

(A) PSN_2687 from NGC7

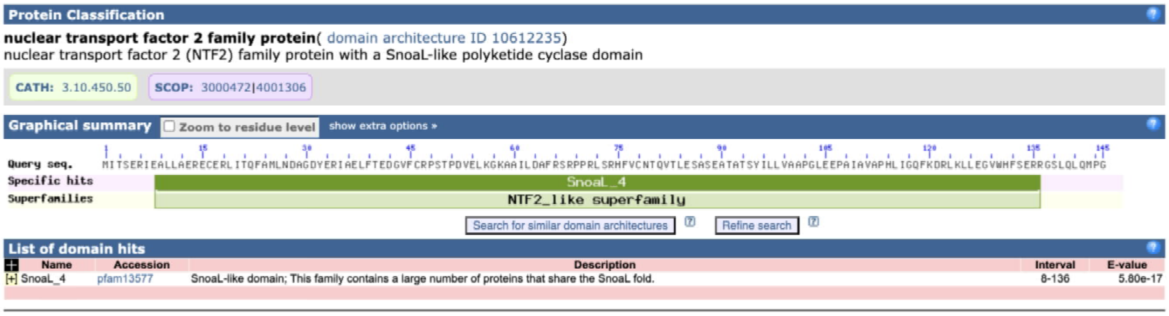

(B) PSN_2688 from NGC7

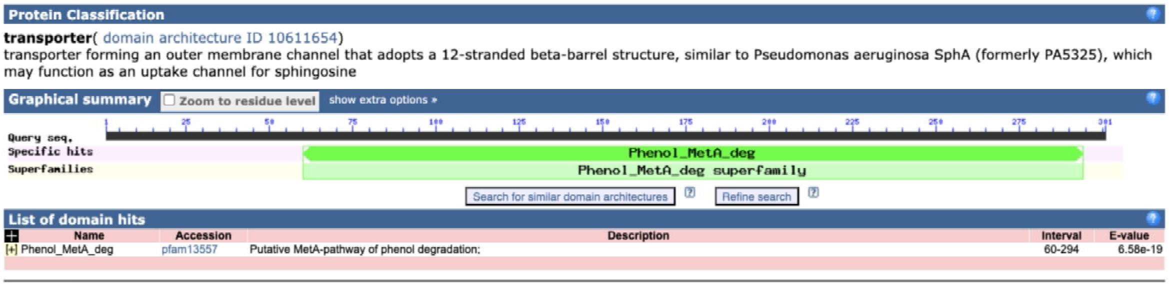

Figure S7. Domain prediction of PSN_2687 (MgaD) and PSN_2688.

(A) The predicted domain of PSN_2687 (MgaD). PSN_2687 is classified in the nuclear transport factor 2 family (domain architecture ID: 10612235) with a SnoaL-like domain (pfam: 13577). Proteins in this family include lyase, isomerase, dehydratase, dehydrochlorinase, and decarboxylase [21]. (B) The predicted domain of PSN_2688. PSN_2688 is classified in the transporter family (domain architecture ID: 10611654) with a Phenol_MetA_deg domain (pfam: 13557). Proteins in this family include SphA, which is predicted to be involved in sphingosine uptake in *Pseudomonas aeruginosa* [22].

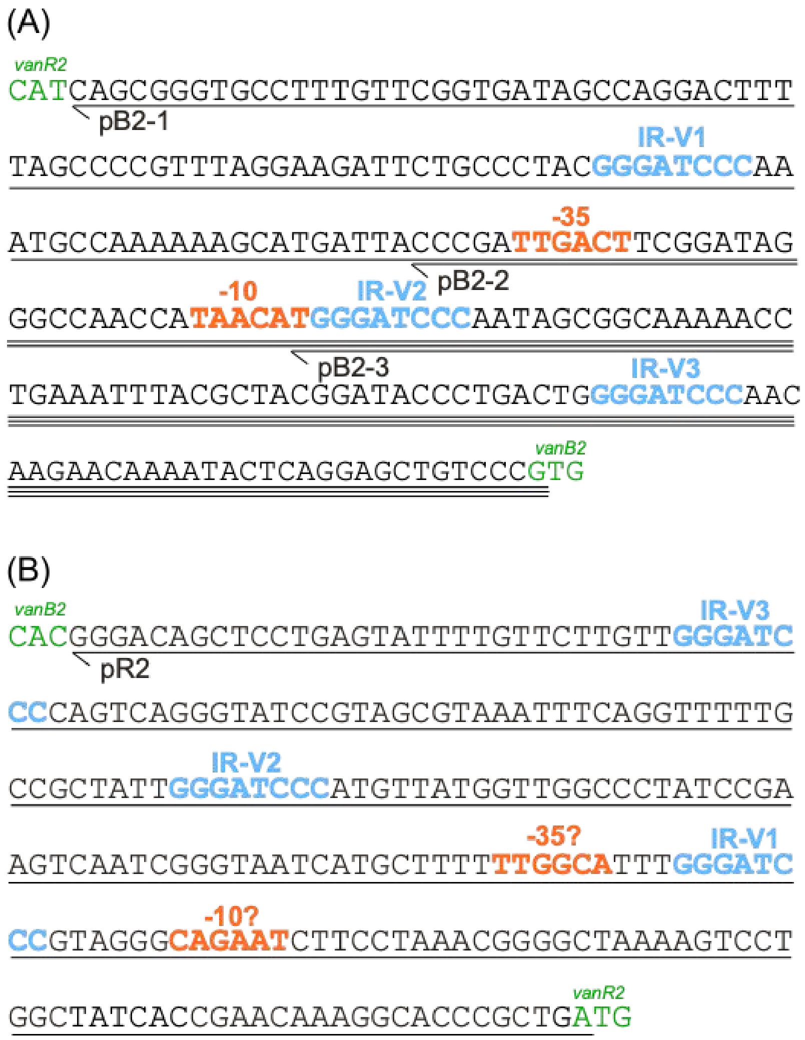

Figure S8. The nucleotide sequence of the intergenic region *vanR2-vanB2.*

Sequences used in the *vanB2* promoter assay (pB2) and *vanR2* promoter assay (pR2), respectively, are underlined. *vanB2* promoter sequences (A) and the putative *vanR2* promoter sequences (B). VanR2 binding sites (IR-V1, IR-V2, and IR-V3), putative -35 and -10 sequences, start and stop codon of *vanB2* and *vanR2* are shown in light blue, orange, and green, respectively.

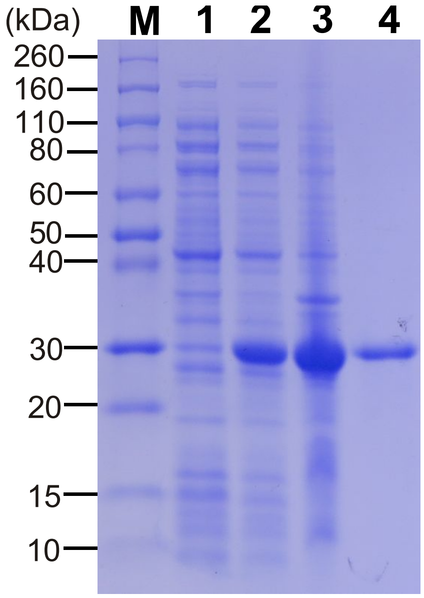

Figure S9. Expression of *vanR2* in *E. coli* and purification of the gene products.

Proteins were separated on SDS-12% polyacrylamide gels and stained with Coomassie Brilliant Blue. Lanes: M, molecular mass markers; 1, soluble fractions (10 μg protein) of *E. coli* BL21(DE3) cells harboring pET-21a(+) (vector); 2 and 3, soluble and insoluble fractions of *E. coli* BL21(DE3) cells harboring pET-vanR2; 4, Purified VanR2 (2 μg protein).

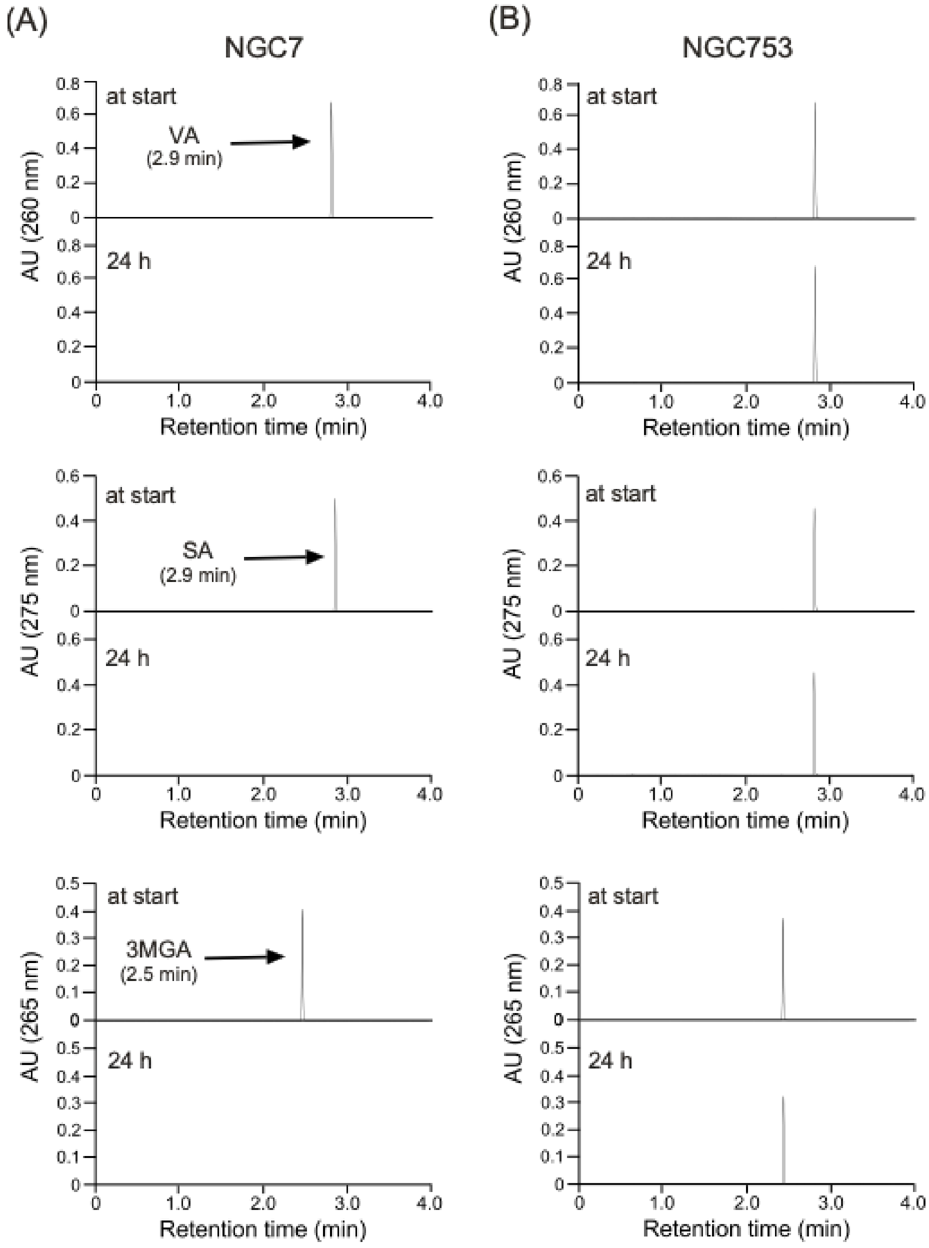

Figure S10. Conversion of VA, SA, and 3MGA by NGC7 and NGC753.

HPLC chromatograms of culture supernatants from NGC7 (A) and NGC753 (B). Chromatograms at 260 nm (top), 275 nm (middle), and 265 nm (bottom) for the detection of VA, SA, and 3MGA are shown. Cells of NGC7 and NGC753 were grown in MMx-3 medium containing 5 mM Glc + VA, 5 mM Glc + SA, and 5 mM Glc + 3MGA. Samples were collected at the start and after 24 h, and then analyzed by HPLC.

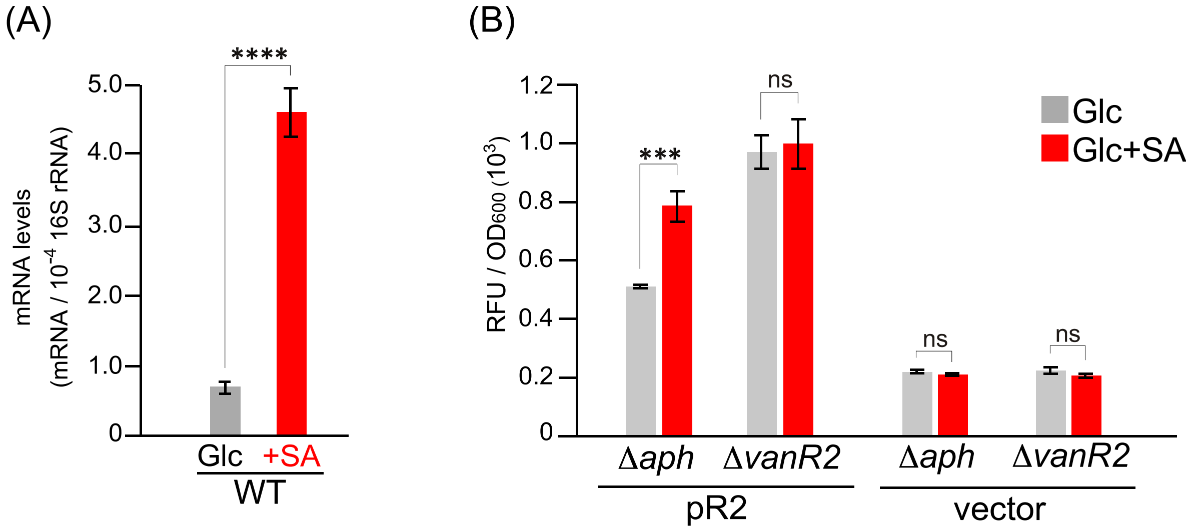

Figure S11. Involvement of VanR2 in its own transcriptional regulation.

(A) qRT-PCR analysis of *vanR2*. The mRNA levels of *vanR2* in NGC7 grown on 5 mM Glc or 5 mM Glc + 5 mM SA were quantified by qRT-PCR. mRNA levels were normalized to 16S rRNA. (B) Promoter analysis of *vanR2*. Promoter activities (RFU/OD_{600}) of Δ*aph* (the wild-type control) and Δ*vanR2* cells harboring pR2, containing the upstream region of *vanR2* or pSEVAmNeon (vector) grown on 5 mM Glc and 5 mM Glc + 5 mM SA. Each value represents the mean ± standard deviation (error bars) from three independent experiments. Asterisks show statistically significant differences between cells grown on Glc and Glc + SA (ns, $p > 0.05$; ***, $p < 0.001$; ****, $p < 0.0001$).

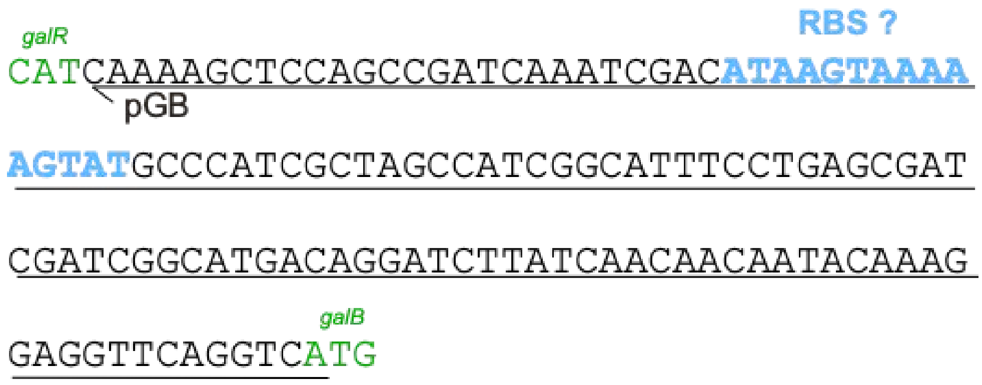

Figure S12. Nucleotide sequence of the intergenic region between *galR* and *galB*.
Sequences that were inserted into the *galB* promoter assay plasmid pGB are underlined. The putative GalR binding site (RBS) and the start codons of *galR* and *galB*, are shown in light blue and green, respectively.

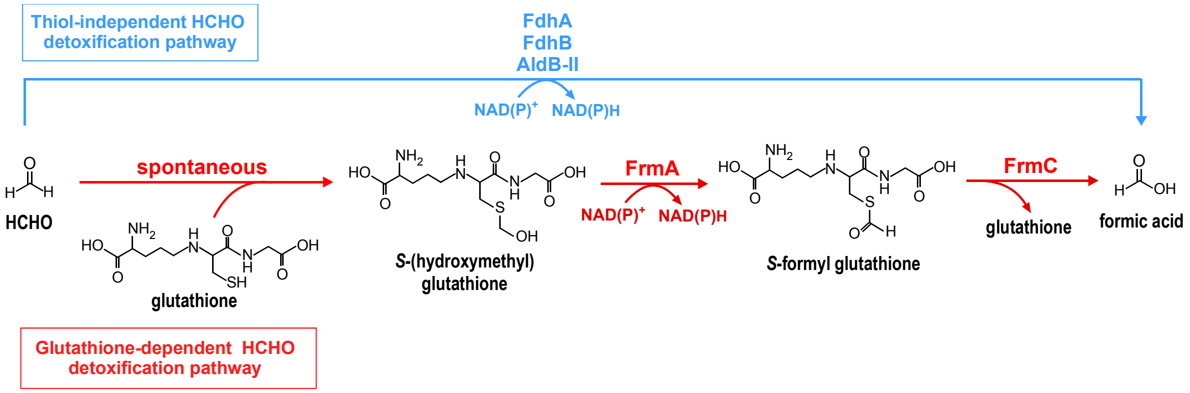

Figure S13. The HCHO detoxification pathways in *P. putida* KT2440.

The thiol-independent HCHO detoxification pathway and the glutathione-dependent HCHO detoxification pathway in *P. putida* KT2440 are indicated by light blue and red, respectively [23]. Enzymes: FdhA and FdhB, HCHO dehydrogenase; AldB-II, alcohol dehydrogenase; FrmA, *S*-(hydroxymethyl)glutathione dehydrogenase; FrmC, *S*-(hydroxymethyl)glutathione hydrolase.

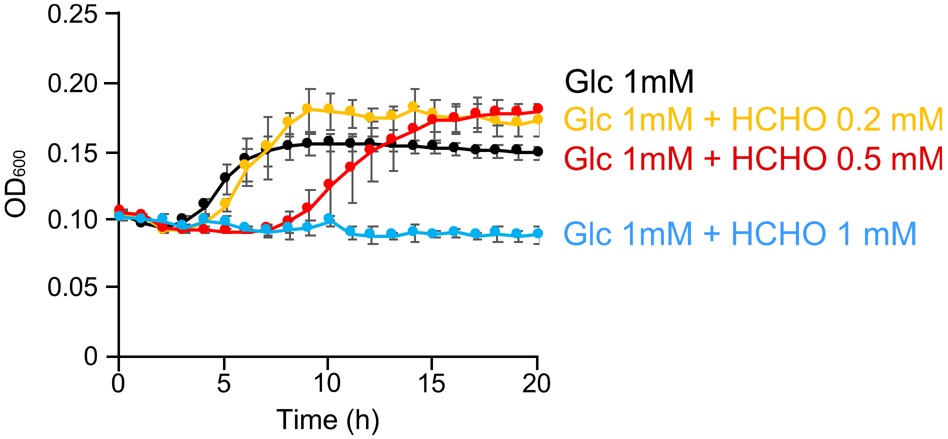

Figure S14. Growth inhibition of NGC7 cells by HCHO.

Growth of NGC7 cells on 1 mM Glc or 1 mM Glc + 0.2, 0.5, and 1 mM HCHO were measured. Cell growth was monitored by measuring the OD_{600}. Each value represents the mean ± standard deviation (error bars) from three independent experiments.

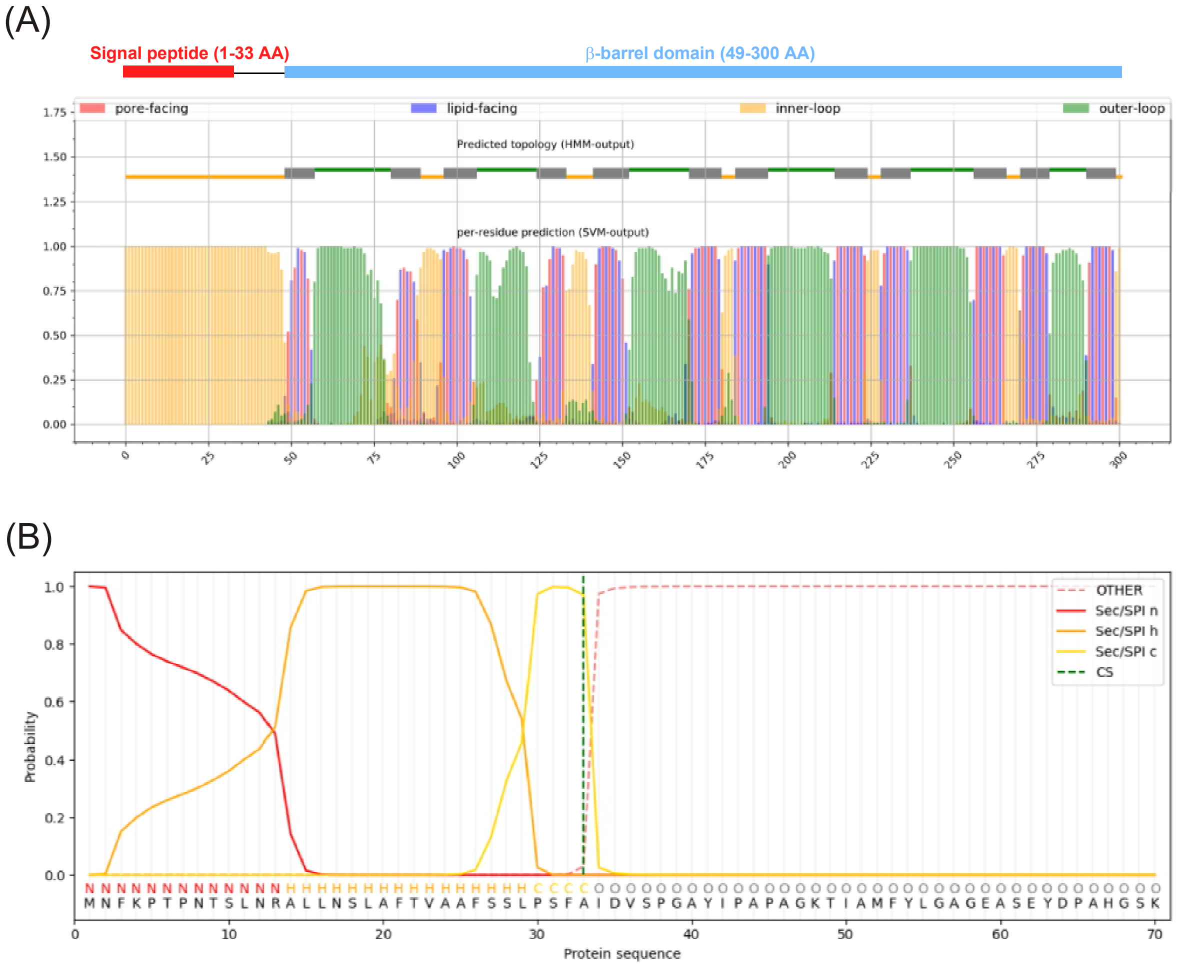

Figure S15. Prediction of transmembrane β-barrel and signal peptide in PSN_2688.

(A) Topology prediction of transmembrane β-barrels in PSN_2688 using BOCTOPUS2 [8]. (B) Prediction of signal peptide in PSN_2688 using SignalP 6.0 [9].

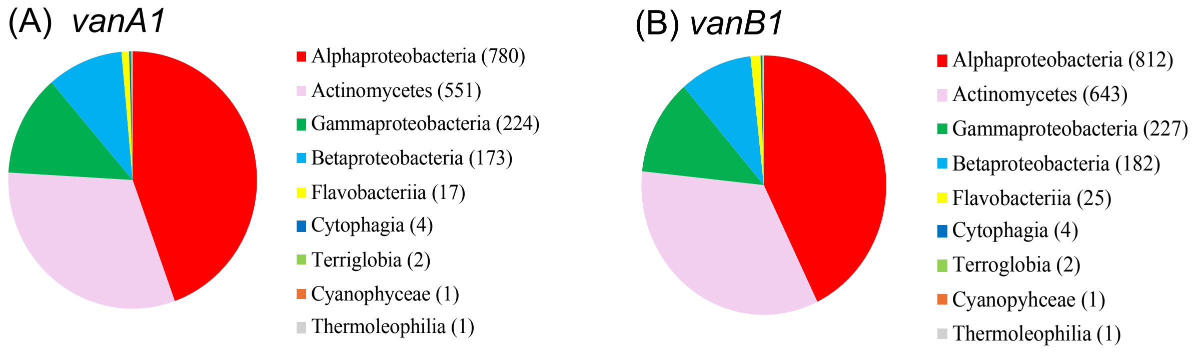

Figure S16. Conservation of *vanA1B1* in Bacteria.

The taxonomic classes in which homologs of *vanA1* (A) or *vanB1* (B) were found. Homologs were defined as genes exhibiting >70% query cover for the amino acid sequence identity of *vanA1* and *vanB1*, as determined by NCBI Protein BLAST program with non-redundant protein sequence database searches, and >50% and >40% sequence identity, respectively. The numbers in parentheses represent the number of organisms possessing homologs in each class. The detailed results of the homology searches for *vanA1* and *vanB1* are provided in Supplemental data sheets 1 and 2, respectively.

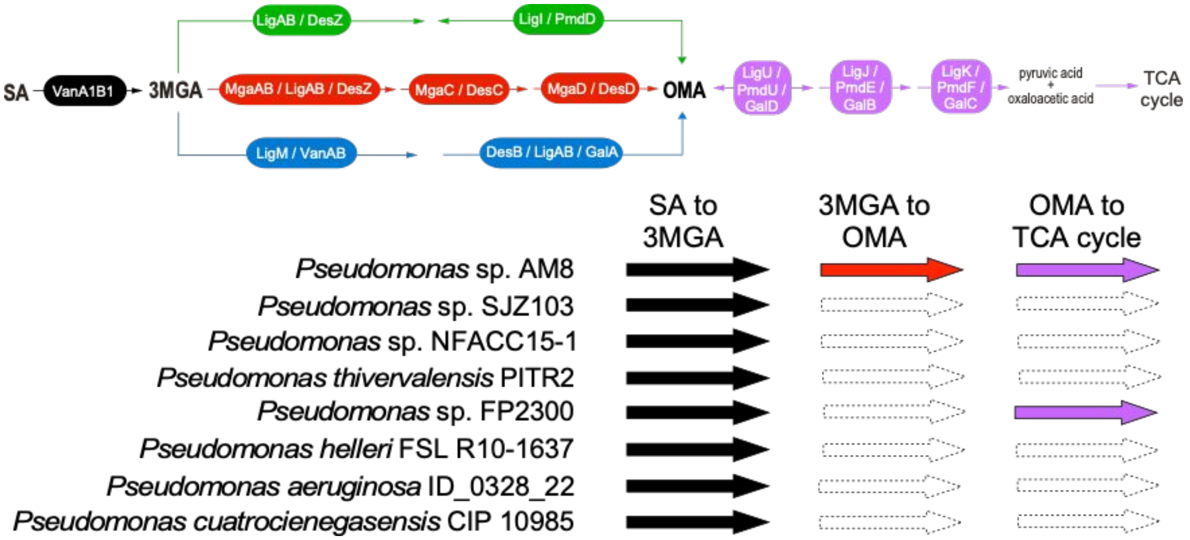

Figure S17. Conservation of SA catabolism genes in *Pseudomonas*.

Conservation of SA catabolic genes (>50% amino acid sequence identity, >70% query coverage) was investigated for all *Pseudomonas* strains containing homologs of *vanA1* and *vanB1*. The enzymes and organisms used in this investigation are listed in Table S6 and supplemental data sheet 4, respectively. The previously reported SA catabolic pathways are illustrated above. Strains in which both *vanA1* and *vanB1* are responsible for converting SA to 3MGA are indicated by black arrows. Among the branched 3MGA to OMA catabolic pathways, strains containing *ligAB*/*desZ* and *ligI*/*pmdD*, *mgaAB*/*ligAB*/*desZ* and *mgaC*/*desC*, or *ligM*/*vanAB* and *desB*/*ligAB*/*galA* are indicated by green, red, and blue arrows, respectively. Strains with *ligU*/*pmdU*/*galD*, *ligJ*/*pmdE*/*galB*, and *ligK*/*pmdF*/*galC*, which are responsible for converting OMA to products that enter the TCA cycle, are indicated by purple arrows. Thus, strains with three arrows possess one of the enzyme-encoding genes for all reaction steps from SA to the TCA cycle.

REFERENCES

(1) Akutsu, M.; Abe, N.; Sakamoto, C.; Kurimoto, Y.; Sugita, H.; Tanaka, M.; Higuchi, Y.; Sakamoto, K.; Kamimura, N.; Kurihara, H. *Pseudomonas* sp. NGC7 as a microbial chassis for glucose-free muconate production from a variety of lignin-derived aromatics and its application to the production from sugar cane bagasse alkaline extract. *Bioresource Technology* **2022**, *359*, 127479. DOI: 10.1016/j.biortech.2022.127479.

(2) Otsuka, Y.; Nakamura, M.; Shigehara, K.; Sugimura, K.; Masai, E.; Ohara, S.; Katayama, Y. Efficient production of 2-pyrone-4,6-dicarboxylic acid as a novel polymer-based material from protocatechuate by microbial function. *Applied Microbiology and Biotechnology* **2006**, *71*, 608-614. DOI: 10.1007/s00253-005-0203-7.

(3) Madeira, F.; Madhusoodanan, N.; Lee, J.; Eusebi, A.; Niewielska, A.; Tivey, A. R.; Lopez, R.; Butcher, S. The EMBL-EBI Job Dispatcher sequence analysis tools framework in 2024. *Nucleic Acids Research* **2024**, *52* (W1), W521-W525. DOI: 10.1093/nar/gkae241.

(4) Katoh, K.; Rozewicki, J.; Yamada, K. D. MAFFT online service: multiple sequence alignment, interactive sequence choice and visualization. *Briefings in Bioinformatics* **2019**, *20* (4), 1160-1166. DOI: 10.1093/bib/bbx108.

(5) Pruesse, E.; Peplies, J.; Glöckner, F. O. SINA: accurate high-throughput multiple sequence alignment of ribosomal RNA genes. *Bioinformatics* **2012**, *28* (14), 1823-1829. DOI: 10.1093/bioinformatics/bts252.

(6) Wang, J.; Chitsaz, F.; Derbyshire, M. K.; Gonzales, N. R.; Gwadz, M.; Lu, S.; Marchler, G. H.; Song, J. S.; Thanki, N.; Yamashita, R. A. The conserved domain database in 2023. *Nucleic Acids Research* **2023**, *51* (D1), D384-D388. DOI: 10.1093/nar/gkac1096.

(7) Johnson, M.; Zaretskaya, I.; Raytselis, Y.; Merezhuk, Y.; McGinnis, S.; Madden, T. L. NCBI BLAST: a better web interface. *Nucleic Acids Research* **2008**, *36* (suppl_2), W5-W9. DOI: 10.1093/nar/gkn201.

(8) Hayat, S.; Peters, C.; Shu, N.; Tsirigos, K. D.; Elofsson, A. Inclusion of dyad-repeat pattern improves topology prediction of transmembrane β-barrel proteins. *Bioinformatics* **2016**, *32* (10), 1571-1573. DOI: 10.1093/bioinformatics/btw025.

(9) Teufel, F.; Almagro Armenteros, J. J.; Johansen, A. R.; Gíslason, M. H.; Pihl, S. I.; Tsirigos, K. D.; Winther, O.; Brunak, S.; von Heijne, G.; Nielsen, H. SignalP 6.0 predicts all five types of signal peptides using protein language models. *Nature Biotechnology* **2022**, *40* (7), 1023-1025. DOI: 10.1038/s41587-021-01156-3.

(10) Minh, B. Q.; Schmidt, H. A.; Chernomor, O.; Schrempf, D.; Woodhams, M. D.; Von Haeseler, A.; Lanfear, R. IQ-TREE 2: new models and efficient methods for phylogenetic inference in the genomic era. *Molecular Biology and Evolution* **2020**, *37* (5), 1530-1534. DOI: 10.1093/molbev/msaa015.

(11) Letunic, I.; Bork, P. Interactive Tree of Life (iTOL) v6: recent updates to the phylogenetic tree display and annotation tool. *Nucleic Acids Research* **2024**, *52* (W1), W78-W82. DOI: 10.1093/nar/gkae268.

(12) Kamada, M.; Yasuta, C.; Higuchi, Y.; Yoshida, A.; Kurnia, I.; Sakamoto, C.; Takeuchi, A.; Osaka, Y.; Muraki, K.; Kamimura, N. Engineering a vanillate-producing strain of *Pseudomonas* sp. NGC7 corresponding to aromatic compounds derived from the continuous catalytic alkaline oxidation of sulfite lignin. *Microbial Cell Factories* **2024**, *23* (1), 1-12. DOI: 10.1186/s12934-024-02590-z.

(13) Bolger, A. M.; Lohse, M.; Usadel, B. Trimmomatic: a flexible trimmer for Illumina sequence data. *Bioinformatics* **2014**, *30* (15), 2114-2120. DOI: 10.1093/bioinformatics/btu170.

(14) Langmead, B.; Salzberg, S. L. Fast gapped-read alignment with Bowtie 2. *Nature Methods* **2012**, *9* (4), 357-359. DOI: 10.1038/nmeth.1923.

(15) Community, G. The Galaxy platform for accessible, reproducible, and collaborative data analyses: 2024 update. *Nucleic Acids Research* **2024**, *52* (W1), W83-W94. DOI: 10.1093/nar/gkae410.

(16) Bradford, M. M. A rapid and sensitive method for the quantitation of microgram quantities of protein utilizing the principle of protein-dye binding. *Analytical Biochemistry* **1976**, *72* (1-2), 248-254. DOI: 10.1016/0003-2697(76)90527-3.

(17) Shinoda, E.; Takahashi, K.; Abe, N.; Kamimura, N.; Sonoki, T.; Masai, E. Isolation of a novel platform bacterium for lignin valorization and its application in glucose-free *cis, cis*-muconate production. *Journal of Industrial Microbiology and Biotechnology* **2019**, *46* (8), 1071-1080. DOI: 10.1007/s10295-019-02190-6.

(18) Studier, F. W.; Moffatt, B. A. Use of bacteriophage T7 RNA polymerase to direct selective high-level expression of cloned genes. *Journal of Molecular Biology* **1986**, *189* (1), 113-130. DOI: 10.1016/0022-2836(86)90385-2.

(19) Schäfer, A.; Tauch, A.; Jäger, W.; Kalinowski, J.; Thierbach, G.; Pühler, A. Small mobilizable multi-purpose cloning vectors derived from the *Escherichia coli* plasmids pK18 and pK19: selection of defined deletions in the chromosome of *Corynebacterium glutamicum*. *Gene* **1994**, *145* (1), 69-73. DOI: 10.1016/0378-1119(94)90324-7.

(20) Martínez-García, E.; Fraile, S.; Algar, E.; Aparicio, T.; Velázquez, E.; Calles, B.; Tas, H.; Blázquez, B.; Martín, B.; Prieto, C. SEVA 4.0: an update of the Standard European Vector Architecture database for advanced analysis and programming of bacterial phenotypes. *Nucleic Acids Research* **2023**, *51* (D1), D1558-D1567. DOI: 10.1093/nar/gkac1059.

(21) Kuatsjah, E.; Zahn, M.; Chen, X.; Kato, R.; Hinchen, D. J.; Konev, M. O.; Katahira, R.; Orr, C.; Wagner, A.; Zou, Y.; et al. Biochemical and structural characterization of a sphingomonad diarylpropane lyase for cofactorless deformylation. *Proceedings of the National Academy of Sciences* **2023**, *120* (4), e2212246120. DOI: 10.1073/pnas.2212246120.

(22) Labauve, A. E.; Wargo, M. J. Detection of Host-Derived Sphingosine by *Pseudomonas aeruginosa* Is Important for Survival in the Murine Lung. *PLoS Pathogens* **2014**, *10* (1), e1003889. DOI: 10.1371/journal.ppat.1003889.

(23) Turlin, J.; Puiggené, Ò.; Donati, S.; Wirth, N. T.; Nikel, P. I. Core and auxiliary functions of one-carbon metabolism in *Pseudomonas putida* exposed by a systems-level analysis of transcriptional and physiological responses. *mSystems* **2023**, *8* (3), e00004-00023. DOI: 10.1128/msystems.00004-23.
